# Human-made vs. AI-generated: how provenance labels drive strategic curation via perceived effort

**DOI:** 10.3389/fpsyg.2026.1840483

**Published:** 2026-06-03

**Authors:** Han Sol Lim, Bong Gyou Lee, Yoon Hi Sung, Chang Won Jung

**Affiliations:** 1 Graduate School of Information, Yonsei University, Seoul, Republic of Korea; 2 Department of Communication, Yonsei University, Seoul, Republic of Korea; 3 Department of Media Communication, Dankook University, Gyeonggi-do, Republic of Korea

**Keywords:** AI disclosure, content provenance, generative AI, human-made labeling, perceived effort, short-form video, strategic curation, user agency

## Abstract

**Introduction:**

This study examined how content provenance labeling on short-form video platforms shapes users’ perceived creator effort, algorithmic curation efficacy, and strategic curation intention. Whereas prior research has largely framed such labels as instruments for risk disclosure or warning, this study reconceptualizes provenance labels as value signals (i.e., attribution cues that signal and assign creative effort) and investigates the psychological mechanisms through which these signals create beliefs about algorithmic intervention and affect subsequent behavioral intentions.

**Methods:**

A between-subjects experiment was conducted with 618 short-form video users using a 3 (label type: human-made vs. AI-generated vs. unlabeled) × 2 (content type: eudaimonic vs. hedonic) factorial design to test labeling effects and the dual-pathway mechanism linking perceived effort with strategic curation.

**Results:**

The AI-generated label significantly reduced perceived effort, whereas the human-made label did not differ from the unlabeled condition, providing empirical evidence for an implicit human-made default assumption. Perceived effort increased strategic curation intentions via both rational and normative pathways. However, the AI-generated label weakened both pathways, producing an asymmetric dual-path effect that systematically undermined users’ willingness to intervene in algorithmic curation.

**Discussion:**

The effort devaluation induced by AI labeling operated as a context-independent heuristic, unaffected by content type or perceived platform degradation. Moreover, an exploratory finding showed that greater algorithmic knowledge was associated with lower intervention intention, suggesting that user agency may be grounded less in technical knowledge than in subjective efficacy beliefs. This implies that future labeling policies should move beyond passive risk disclosure toward a human-centered value-certification framework that foregrounds human creativity and effort.

## 1 Introduction

Recent public discourses such as the “Dead Internet Theory” and concerns over “AI slop” reflect a growing anxiety that algorithmically mass-produced, mechanical content is encroaching on human communication spaces (McMillan, 2023; Walter, 2025; Tenbarge, 2025; Muzumdar et al., 2025). The unrestrained diffusion of generative artificial intelligence (GenAI)-made content has intensified information overload and user fatigue on short-form platforms while heightening uncertainty about what constitutes genuine human creation. This uncertainty reinforces users’ sense of powerlessness and feelings that it has become increasingly difficult to have one’s own value preferences reflected by algorithmic systems (DeVito, 2021; Yang et al., 2024).

In this environment, users’ central concerns have expanded beyond the question of what to watch to how to respond to content in ways that shape their informational environment. Prior research suggests that users strategically engage with preferred content to “teach” the algorithm while simultaneously employing defensive tactics (e.g., withholding clicks or reactions) to prevent their feeds from being polluted by unwanted material (Siles et al., 2020; Swart, 2021; Haupt et al., 2023). Refraining from interaction can therefore be just as deliberate and calculated as actively pursuing content, insofar as both are aimed at preventing algorithmic mislearning. This article conceptualizes intentional modulation or restraint in responses to influence future recommendation outcomes as strategic curation. Importantly, this positions users as active agents who seek to manage and curate their feeds (Yuan et al., 2025). For strategic curation to function effectively, users also require cues that allow them to rapidly assess the value of content.

In response to the challenges posed by the rise of AI-generated content, platform companies have introduced transparency policies that attach labels to such materials. However, current approaches largely frame labeling as a risk-warning mechanism designed to signal the presence of deepfakes or misinformation (Li and Yang, 2024; Wittenberg et al., 2025). In entertainment-oriented, short-form environments, where users must make intuitive judgments under severe time constraints, provenance labels are more likely to function as interpretive cues that immediately orient how content is understood (Sundar, 2020; Gamage et al., 2025; Chen et al., 2025). In this process, labels may operate as critical signals that shape how users attribute human contribution and creative effort to content. If provenance information is a starting point for value judgment, then within social media environments—where human creation has long been assumed as an implicit default—labels may either reinforce human-centered standards or, alternatively, reveal mechanical generation and thereby operate as a penalty that devalues content. To date, however, empirical research has not consistently established whether provenance labels in short-form environments function primarily as warning disclosures, as value signals, or as cues that shape strategic user responses.

Against this backdrop, this paper advances existing transparency debates by conceptualizing provenance labels as value signals that reveal creators’ efforts and examining the psychological mechanisms through which such signals activate strategic curation among users. In doing so, the study shifts the focus from disclosure as mere risk communication to disclosure as a cue for value attribution and algorithm-oriented user response. This study focused on two distinct pathways through which effort attribution shapes user behavior. The first was a normative pathway, in which the norm of reciprocity motivates users to reward others’ time and labor when more effort is perceived (Morales, 2005; Siles et al., 2020). The second was a rational pathway, in which perceived effort (PE) fostered curation efficacy—the belief that one’s own responses can meaningfully influence the system—and thereby strengthened strategic action (Siles et al., 2020; Swart, 2021; Haupt et al., 2023). The critical question was whether provenance labels operated as a positive mechanism that elevated effort perception and efficacy or whether they instead triggered effort discounting and efficacy erosion, suppressing strategic curation.

To address this question, this study empirically examined how content provenance labels (human-made vs. AI-generated vs. unlabeled) influenced strategic curation intention (SCI) through PE and algorithmic curation efficacy (ACE) in short-form video environments. Using the unlabeled condition as a baseline, the analysis tested the potential asymmetry of labeling effects and investigated whether contextual factors—such as casual consumption orientations (hedonic vs. eudaimonic) and perceived platform pollution (PPP)—moderated these value judgments. The study further examined whether algorithmic literacy (AL) facilitated the translation of efficacy into action or, paradoxically, amplified user helplessness. This positioned AI provenance labeling as a central mechanism through which users enact value judgments and strategic curation, that is, intentional user responses aimed at shaping future recommendation outcomes, thereby offering a theoretical foundation for human-centered platform design.

## 2 Theoretical background

### 2.1 Content provenance signals and labeling in the era of GenAI

Discussions about GenAI labeling have largely centered on technical transparency regarding content origin and production mode, with an emphasis on mitigating the risks of misinformation and deepfakes (National Institute of Standards and Technology, 2023; Weidinger et al., 2023; Li and Yang, 2024; Wittenberg et al., 2024; European Union, 2024; Gamage et al., 2025). These approaches implicitly assume human creation as the default and treat AI labels primarily as risk-oriented disclosure cues. However, rapid advances in GenAI are increasingly challenging assumptions of default human creation. Distinctions between human and machine authorship have grown ambiguous, and binary labeling schemes risk losing discriminatory power or increasing user confusion (Wittenberg et al., 2024; Jung et al., 2025; Nserat, 2025; Gamage et al., 2025). The inconsistent implementation of provenance labels across platforms further undermines their reliability as evaluative cues (Bond, 2023; Goldman, 2024; TikTok, 2025).

Despite these shifts, most policy and research frameworks continue to conceptualize labeling as a protective disclosure mechanism for informative or advertising contexts, with limited attention to entertainment-oriented short-form platforms or how provenance information shapes users’ value judgments and behaviors (Toff and Simon, 2025; Park et al., 2024; Huh et al., 2025; Baptista et al., 2025; Chen et al., 2025; Heimstad et al., 2025). Recent initiatives like Not by AI reflect a growing societal demand for clear distinctions between human creation and automated output, prompting a shift from disclosure-based labeling toward value-oriented certification logics that foreground creative process and provenance (Van Doorn and Verhoef, 2011; Not By AI, 2023; Li and Yang, 2024; Bonos, 2025; Wittenberg et al., 2025). From a signaling theory perspective, such labels can be understood as observable cues that help users infer otherwise unobservable production attributes under information asymmetry, allowing users to infer creative value and human involvement from otherwise opaque outputs (Spence, 1973; Connelly et al., 2011).

### 2.2 Signaling theory and the handmade effect

Although signaling theory explains how labels reduce information asymmetry by making otherwise hidden production attributes observable, it does not fully account for why certain attributes, such as human involvement or creative effort, carry greater evaluative weight than others. The handmade effect from consumer psychology offers a useful framework for explaining this evaluative weight (Fan, 2025). The handmade effect refers to the tendency for consumers to attach greater value to products perceived as the result of human craftsmanship and care than to functionally equivalent machine-made counterparts, even when objective quality is held constant. Fuchs et al. (2015) demonstrated this effect across product categories, showing that perceived human effort and care drive higher evaluations independent of objective quality. Extending this logic to digital content, Bellaiche et al. (2023) showed that human-created labels increase aesthetic evaluation and emotional impact relative to AI-generated labels by activating inferences about creator effort.

Building on this evaluative logic, human-made and AI-generated labels can be understood as carrying distinct semantic implications for users. A human-made label invokes inferences associated with embodied human creativity, sustained practice, deliberate intention, and visible labor, signaling that the output is the product of a maker who invested time, skill, and care (Fuchs et al., 2015; Bellaiche et al., 2023). An AI-generated label, by contrast, frames the output as the result of automated computational processes that bypass these traditional markers of creative labor, cueing users to discount the very markers of creative effort that the human-made label affirms (Magni et al., 2024; Messer, 2024; Heimstad et al., 2025).

### 2.3 Key constructs in the present study

#### 2.3.1 Perceived effort

The divergent semantic implications of human-made and AI-generated labels are unlikely to arise directly from the labels themselves; rather, they operate through users’ inferences about the creative labor that the content embodies. PE captures precisely such inferences, referring to users’ subjective judgments about the time, energy, physical labor, and cognitive commitment invested in producing content, independent of its objective quality (Kruger et al., 2004). PE is particularly relevant in short-form environments, where rapid scrolling, brief exposure times, and limited contextual information about creators leave users with few direct means to assess how a piece of content was actually produced. Under such conditions, effort attribution becomes an inferential rather than observational process, and users tend to rely on whatever cues are made available to them when forming evaluative judgments about creative labor (Judge et al., 2020; Magni et al., 2024; Heimstad et al., 2025).

Consumer psychology has long shown that PE functions as a heuristic cue in evaluation. The labor illusion, whereby outcomes associated with greater inferred labor receive higher value assessments regardless of objective quality, illustrates how cues about production effort can shape users’ value judgments even when the production process is not directly observed (Buell and Norton, 2011). The challenge of inferring effort from output alone is amplified by GenAI, which enables outputs that previously required prolonged training and skill to be produced rapidly and at scale, further blurring the markers that might otherwise distinguish genuine human labor from automated production (Magni et al., 2024; Messer, 2024; Rix et al., 2025). Under these conditions, provenance labels can step in as substitute cues that supply the effort information users would otherwise infer from contextual production traces.

#### 2.3.2 Algorithmic curation efficacy

ACE can be understood as a platform-specific form of psychological empowerment in algorithmically curated environments. Psychological empowerment captures an active motivational state in which individuals perceive their actions as competent, self-determined, meaningful, and capable of producing impact within a particular environment (Spreitzer, 1995). Prior information systems research has similarly applied psychological empowerment to digital system use, showing that system-specific empowerment can motivate proactive user behavior within technology-mediated environments (Kang et al., 2017). Applied to recommender systems, ACE reflects users’ perceived capacity to intervene meaningfully in a partially opaque platform environment through identifiable behavioral signals.

#### 2.3.3 Strategic curation intention

SCI refers to users’ deliberate willingness to send behavioral signals such as liking, saving, or sharing in order to influence algorithmic learning and future recommendations (Siles et al., 2020). Dual-process theories of cognition hold that judgments and behavioral tendencies are commonly shaped by two qualitatively distinct systems: an intuitive, automatic system that responds rapidly on the basis of affective and normative cues, and a deliberative, controlled system that engages in goal-directed reasoning about expected outcomes (Evans and Stanovich, 2013). Applied to strategic curation, this distinction suggests that engagement may reflect either an instrumental judgment that users’ responses can shape future recommendations or a normative judgment that effortful content deserves supportive engagement.

Prior research has conceptualized user–algorithm interaction as a signaling process, in which users strategically select behaviors to optimize future recommendations (Bucher, 2017; Haupt et al., 2023). Within this framework, users are not merely passive recipients of algorithmic outputs, but active curators who attempt to shape their digital environments through intentional engagement. Although liking, saving, subscribing, or exploring related content overlap with conventional engagement indicators in social media research (Khan, 2017), the present study distinguishes strategic curation from generic engagement by emphasizing its underlying purpose of guiding algorithmic learning.

#### 2.3.4 Perceived platform pollution

PPP refers to users’ subjective evaluation of short-form environments as becoming degraded due to the proliferation of AI-generated content, which is linked to weakened authenticity and repetitive algorithmic recommendations. Rather than reflecting technical malfunction or objectively measured content quality, this concept captures users’ holistic perception that their informational environment has become cluttered and unreliable (Shin, 2022; Yang et al., 2024). Contemporary discourses reflect this phenomenological sense of environmental degradation (Walter, 2025; Muzumdar et al., 2025).

#### 2.3.5 Algorithmic literacy

AL refers to users’ awareness of how recommendation systems collect and process behavioral data to generate personalized content, though it stops short of technical or expert-level knowledge (Eslami et al., 2015; Yang et al., 2024). Prior research has conceptualized AL as a causal understanding of data flows or a cognitive map of system functioning, identifying it as an important antecedent of user behavior (Gran et al., 2021; Zarouali et al., 2021).

## 3 Hypothesis development

**Figure 1:**
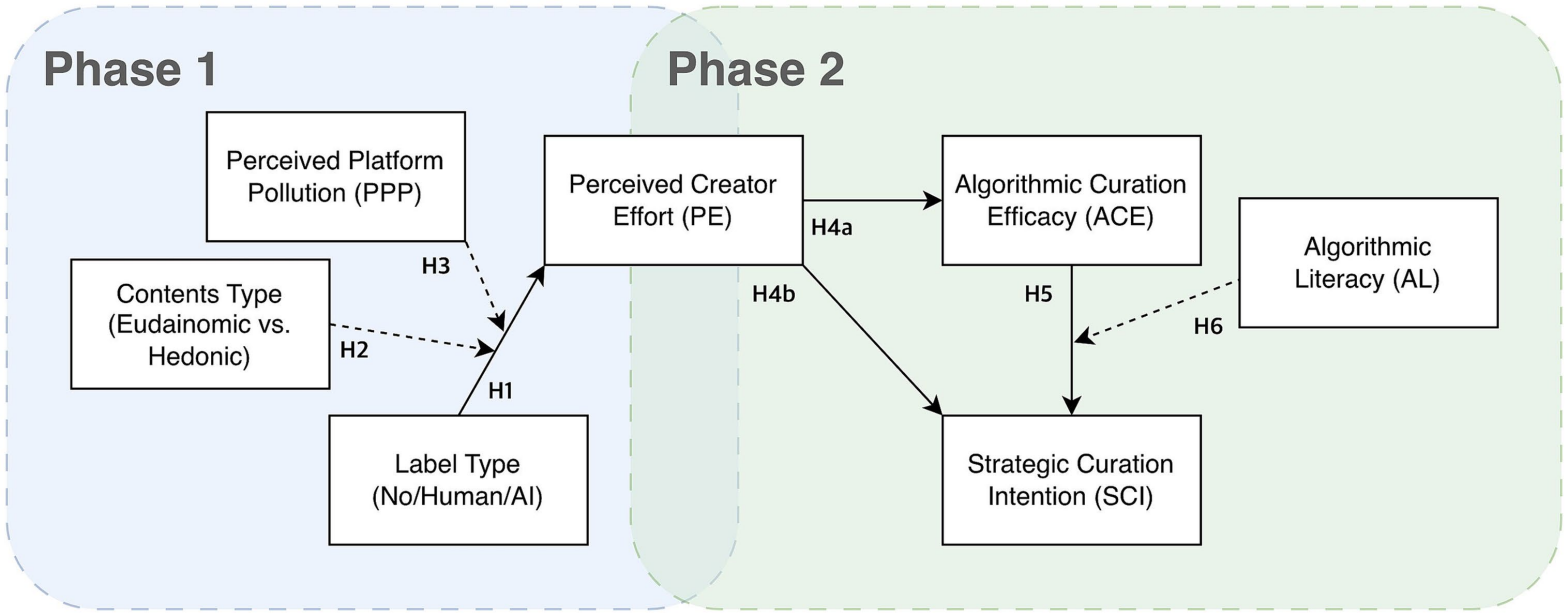
Research model.

Building on the constructs and theoretical foundations laid out in Chapter 2, this chapter develops the hypotheses of the present study. The proposed model conceptualizes labeling effects as operating through two sequential phases: value signaling (Phase 1) and behavioral translation (Phase 2). Phase 1 examines whether provenance labels function as value-signaling cues by influencing PE and whether this effect varies across content types and perceived platform conditions. Phase 2 examines how PE translates into SCI through two distinct pathways and identifies the cognitive condition under which efficacy beliefs are converted into intentional intervention. The overall research model is summarized in Figure 1.

### 3.1 Phase 1: value signaling

#### 3.1.1 Provenance labels and perceived effort

Building on this conceptualization of PE, provenance labels may shape whether users attribute a given output to human skill or algorithmic automation. A “human-made” label encourages users to interpret content as the result of embodied human skill and sustained practice, whereas an “AI-generated” label frames identical outcomes as the automated products of algorithmic computation (Rix et al., 2025). Consistent evidence supports this divergence: human-created labels increase PE, perceived artistry, and willingness to pay even for visually identical stimuli (Bellaiche et al., 2023; Rix et al., 2025; Sikhondze et al., 2025), whereas AI involvement is frequently interpreted as signaling reduced effort across creative and interpersonal domains (Liu et al., 2024).

These effects, however, have rarely been examined in short-form environments, where content is often perceived as originating from individual creators, influencers, or user-generated production. In such environments, unlabeled content is likely to be interpreted through a residual human-authorship assumption unless explicit cues indicate otherwise. Given this default assumption, the two labels are unlikely to carry equivalent informational value. Because a “human-made” label largely confirms what users already presume, its informational gain is limited; in contrast, an “AI-generated” label discloses a deviation from the default and is therefore expected to function as the primary trigger of effort devaluation. Accordingly, provenance labels are predicted to alter PE asymmetrically, with the AI-generated label producing a downward shift relative to the unlabeled baseline while the human-made label produces no comparable upward shift (H1).

*H1.* Content provenance labeling will asymmetrically affect perceived effort, with the AI-generated label reducing perceived effort relative to the unlabeled condition and the human-made label showing no significant difference from the unlabeled condition.

#### 3.1.2 Content type as a boundary condition

Short-form platforms predominantly encourage entertainment-oriented consumption characterized by immediacy and immersion (Yan et al., 2023; Luo et al., 2025; Chen et al., 2025). Within media psychology, entertainment experiences are commonly distinguished between hedonic content, which prioritizes sensory pleasure and enjoyment, and eudaimonic content, which emphasizes meaning, emotional resonance, and reflective engagement (Oliver and Raney, 2011). Short-form users therefore consume both pleasure-oriented content (e.g., dance footage, comedy clips) and meaning-oriented content that highlights craftsmanship, empathy, or creative intention.

These two content types generate systematic differences in information processing and value judgment. Hedonic content directs attention toward experiential outcomes, leading users to prioritize enjoyment over how the content was produced (Rae, 2024). Consistent with the heuristic–systematic model, such low-effort processing environments favor outcome-based evaluation and reduce the weight that users assign to peripheral attribution cues such as provenance labels (Gamage et al., 2025).

In contrast, eudaimonic content foregrounds authenticity, intention, and reflection, encouraging users to attend to the production process and the creator (Oliver and Raney, 2011; Bellaiche et al., 2023). Psychological essentialism holds that people intuitively believe that the value of an object resides in unobservable qualities tied to its origin and the intentions of its maker, rather than in its surface features alone (Newman and Bloom, 2012). From an essentialist perspective, this increases sensitivity to cues that signal human intention and effort, making provenance information more central to value judgment (Sikhondze et al., 2025). Provenance labels, which directly mark whether such human intention is present, are therefore expected to produce a larger difference in perceived effort between human-made and AI-generated labels under eudaimonic content than under hedonic content (H2).

*H2.* The difference in perceived effort between human-made and AI-generated labels will be larger for eudaimonic content than for hedonic content.

#### 3.1.3 Platform pollution as an environmental boundary condition

The present study conceptualizes PPP as an environmental boundary condition that shapes how provenance labels are interpreted. Drawing on the principle of scarcity, prior research suggests that when a valued resource becomes rare or harder to find, its symbolic and evaluative significance increases (Griskevicius et al., 2009). This logic extends to information environments, where the perceived rarity of authentic human creation can elevate its evaluative weight relative to outputs perceived as automated. Accordingly, as users perceive their feeds to be increasingly saturated with AI-generated content, they may become more sensitive to signals that distinguish human-created content as relatively scarce and authentic (Brüns and Meißner, 2024; Muzumdar et al., 2025).

In short-form environments where users perceive a high level of platform pollution, provenance labels are expected to function as particularly salient cues that help users differentiate authentic human effort from automated production within a degraded informational environment. Accordingly, the difference in perceived effort between human-made and AI-generated labels is expected to be more pronounced among users with higher levels of PPP than among users with lower levels (H3).

*H3.* The difference in perceived effort between human-made and AI-generated labels will be larger among users with higher levels of PPP.

### 3.2 Phase 2: behavioral translation

#### 3.2.1 Perceived effort, algorithmic curation efficacy, and strategic curation

Even if provenance labels shape PE, how such effort attributions translate into users’ actual behavioral intentions requires separate explanation. This study draws on dual-process logic to argue that PE shapes SCI through two parallel pathways rather than a single mechanism. The first is a rational pathway in which PE indirectly motivates SCI by strengthening users’ perceived agency in algorithmic environments. The second is a normative pathway in which PE intuitively activates reciprocity-based motivation and translates into engagement as a form of reward for perceived creative labor (Morales, 2005). Each pathway is grounded in a distinct theoretical tradition and is expected to operate concurrently rather than in competition.

The rational pathway operates through a deliberative inference about the value and consequence of one’s behavioral signal. While self-efficacy theory holds that beliefs about one’s ability to influence outcomes are important drivers of behavioral intentions (Bandura, 1977), action in algorithmic environments requires more than a general belief in personal capability. Users are more likely to act strategically when they perceive their actions as self-directed, contextually meaningful, and capable of producing impact within a specific system. This broader sense of agency resonates with psychological empowerment theory, which conceptualizes empowerment as an active motivational state grounded in perceived competence, self-determination, meaning, and impact within a given action context (Spreitzer, 1995). In algorithmically curated environments, users do not directly control recommendation systems, but they may still perceive engagement behaviors such as liking, saving, or sharing as signals through which they can guide future recommendations. Prior research suggests that users often develop folk theories about how platforms learn from behavioral data, while also recognizing that not all forms of engagement carry equal informational weight (Siles et al., 2020; DeVito, 2021; Dogruel, 2021). When content signals substantial creator effort, users may perceive it as more valuable, authentic, and worthy of algorithmic reinforcement (Messer, 2024; Heimstad et al., 2025). Consequently, they may view their own engagement with such content not merely as an expression of preference, but as a meaningful intervention in the recommendation environment (Haupt et al., 2023). In this sense, PE elevates the perceived value of one’s behavioral signal, which in turn raises ACE, the belief that one’s responses can meaningfully shape recommendation outcomes (H4a).

*H4a.* Perceived creator effort will have a positive effect on ACE.

The normative pathway, by contrast, draws on the norm of reciprocity, a fundamental social rule that obligates individuals to reward those who have invested effort or resources on their behalf (Gouldner, 1960; Morales, 2005). When users perceive substantial creator effort, this norm is intuitively activated and generates a motivation to acknowledge that effort, even in the absence of any direct relationship with the creator. In algorithmically curated short-form environments, where users cannot reciprocate through interpersonal exchange, this motivation is instead expressed through engagement behaviors such as liking, saving, or sharing, which serve as platform-mediated forms of reward (Bucher, 2017; Khan, 2017; Messer, 2024; Heimstad et al., 2025). This pathway is driven by intuitive judgments that effort deserves recognition rather than by deliberation about algorithmic consequences (Buell and Norton, 2011), which suggests that PE may exert a direct effect on SCI without being mediated by efficacy beliefs (H4b).

*H4b.* Perceived creator effort will have a direct positive effect on SCI.

This empowerment-based conceptualization clarifies why ACE should translate into SCI. Strategic curation requires users to convert ordinary engagement options into deliberate attempts to train, redirect, or refine the recommendation system. Users with higher ACE are more likely to regard their platform behaviors as consequential rather than merely reactive, because they believe that the system can register these behaviors as meaningful inputs. As a result, they should be more willing to use engagement behaviors strategically to influence the kinds of content recommended to them in the future. Accordingly, ACE is expected to positively predict SCI (H5).

*H5.* ACE will positively affect SCI.

#### 3.2.2 Algorithmic literacy as a cognitive boundary condition

In the present study, AL was conceptualized as a cognitive condition that enables ACE to translate into SCI. Whereas ACE reflects a belief that one’s responses can influence recommendation outcomes, AL captures users’ understanding of how those responses are incorporated into system processes. AL is therefore expected to play a more relevant role at the point where motivation is converted into targeted behavior than at the point where motivation is initially formed.

Even without full technical comprehension, subjective causal understanding is argued to be sufficient for strategic action (DeVito, 2021; Swart, 2021). Accordingly, users with higher AL were expected to interpret their responses as meaningful inputs to the system rather than as mere expressions of affect, thereby facilitating behavioral translation (Shin, 2020; Sundar, 2020; Zarouali et al., 2021; Yang et al., 2024). Based on this reasoning, AL was expected to moderate the positive relationship between ACE and SCI, such that this relationship would be strengthened at higher levels of AL (H6).

An alternative possibility, however, is that greater algorithmic understanding may dampen rather than amplify behavioral translation. Users with higher AL more clearly recognize the scale, opacity, and asymmetry of recommendation systems, which may foster algorithmic skepticism, fatigue, or resignation rather than empowerment (DeVito et al., 2017; Swart, 2021; Yang et al., 2024). When users perceive the system as too complex or too entrenched to be meaningfully shaped by individual behavior, even strong efficacy beliefs may fail to translate into intentional intervention. The present study nonetheless sets the facilitation perspective as its primary prediction, as it is more consistent with the action-oriented logic of self-efficacy theory and with prior evidence that literate users more often engage in strategic adaptation than in disengagement (Bandura, 1977; Siles et al., 2020; DeVito, 2021). The competing literacy paradox perspective remains a theoretically plausible alternative.

*H6.* The positive effect of ACE on SCI will be strengthened as AL increases.

## 4 Materials and methods

### 4.1 Experimental design

This study employed a 3 (label type: “human-made” vs. “AI-generated” vs. unlabeled) × 2 (content type: eudaimonic vs. hedonic) between-subjects factorial design yielding six experimental conditions. Both factors were manipulated between subjects. Participants were randomly assigned to one condition, viewed a single video stimulus, and completed the survey. The between-subjects design was adopted to avoid carryover effects such as fatigue, learning, and comparative judgment bias. This enabled a clear assessment of participants’ immediate responses to provenance labeling (Jakesch et al., 2019; Heimstad et al., 2025; Shibuya et al., 2025).

### 4.2 Participants and sampling

Participants were adult internet users residing in South Korea with prior experience with short-form video platforms (e.g., YouTube Shorts, Instagram Reels, TikTok). Data were collected through an online experimental survey administered by a professional panel provider (Korea Research), and participants received modest compensation. South Korea was selected as the research context due to its rapid diffusion of GenAI services, heightened public awareness of AI-related issues, and advanced policy discussions on AI transparency and regulation (Rashid, 2026). These conditions made it a suitable setting for examining labeling effects.

Stratified quota sampling based on gender and age was employed to enhance sample balance, while random assignment ensured internal validity. Consistent with prior experimental research (Longoni et al., 2019; Sikhondze et al., 2025), the target sample size was approximately 100 participants per condition (*N* ≈ 600). The final sample consisted of 618 valid responses (Valid *N* = 618).

### 4.3 Experimental stimuli

#### 4.3.1 Control and validation strategy

To minimize any exogenous influences’ risk to internal validity, all stimuli were presented as 9:16 vertical short-form videos featuring hands as the sole visual subject, with no facial features shown. This design eliminated potential biases related to facial appearance, gender, or ethnicity while leveraging hands as a diagnostic cue commonly used by users to assess authenticity in visual content (Ivan, 2025). Stimulus equivalence was further ensured to prevent visual quality from confounding labeling effects. Given recent advances in GenAI that enable increasingly realistic visual outputs (Heimstad et al., 2025), all videos were selected to be high-resolution and free from artifacts or unnatural motion. This approach ensured that the production mode could not be inferred from visual cues alone, allowing the study to isolate the effect of provenance labeling on PE and value evaluation. Crucially, all experimental conditions used the same human-created video, differing only in label presentation. As a result, any observed differences in the dependent variables can be attributed exclusively to the signal conveyed by the label, rather than intrinsic differences in content quality.

#### 4.3.2 Stimulus selection and pretest

Experimental stimuli were selected with a shared focus on the hands while ensuring clear contrasts in experiential orientation and information processing style. Following the distinction proposed by Oliver and Raney (2011), finger tutting and finger dance videos were selected for the hedonic condition to emphasize rhythmic dexterity and immediate sensory enjoyment, whereas handwriting and calligraphy videos were chosen for the eudaimonic condition to foreground reflective meaning and emotional resonance conveyed through manual skill and expression.

Candidate videos were collected from major short-form platforms using these criteria. To minimize extraneous cues, all videos featured hands as the primary visual focus, excluded faces and complex backgrounds, and were produced solely by human creators without AI involvement. Three candidate videos were identified for each content type, yielding six preliminary stimuli.

A pretest evaluation was conducted by an expert panel consisting of the research team and six scholars specializing in media psychology. Using the adapted measures of hedonic qualities, eudaimonic qualities, and PE, the panelists assessed the suitability of each video. One video per content type was selected for the final stimuli, ensuring clear differentiation between conditions while maintaining equivalence in video quality, length, and baseline PE.

#### 4.3.3 Factor design

##### 4.3.3.1 Factor a: content type

###### 4.3.3.1.1 Eudaimonic condition

In the eudaimonic condition, the stimulus was an approximately 15-s video depicting an artist carefully transcribing a reflective monologue (e.g., “I will keep drawing…”) with a fountain pen. The slow pace of the handwriting and the visible absorption of ink into the paper were selected for their ability to foreground time, care, and dedication, thereby enhancing PE. The reflective narrative content was intended to elicit aesthetic appreciation and emotional resonance, ensuring the validity of the stimulus as eudaimonic.

**Figure 2:**
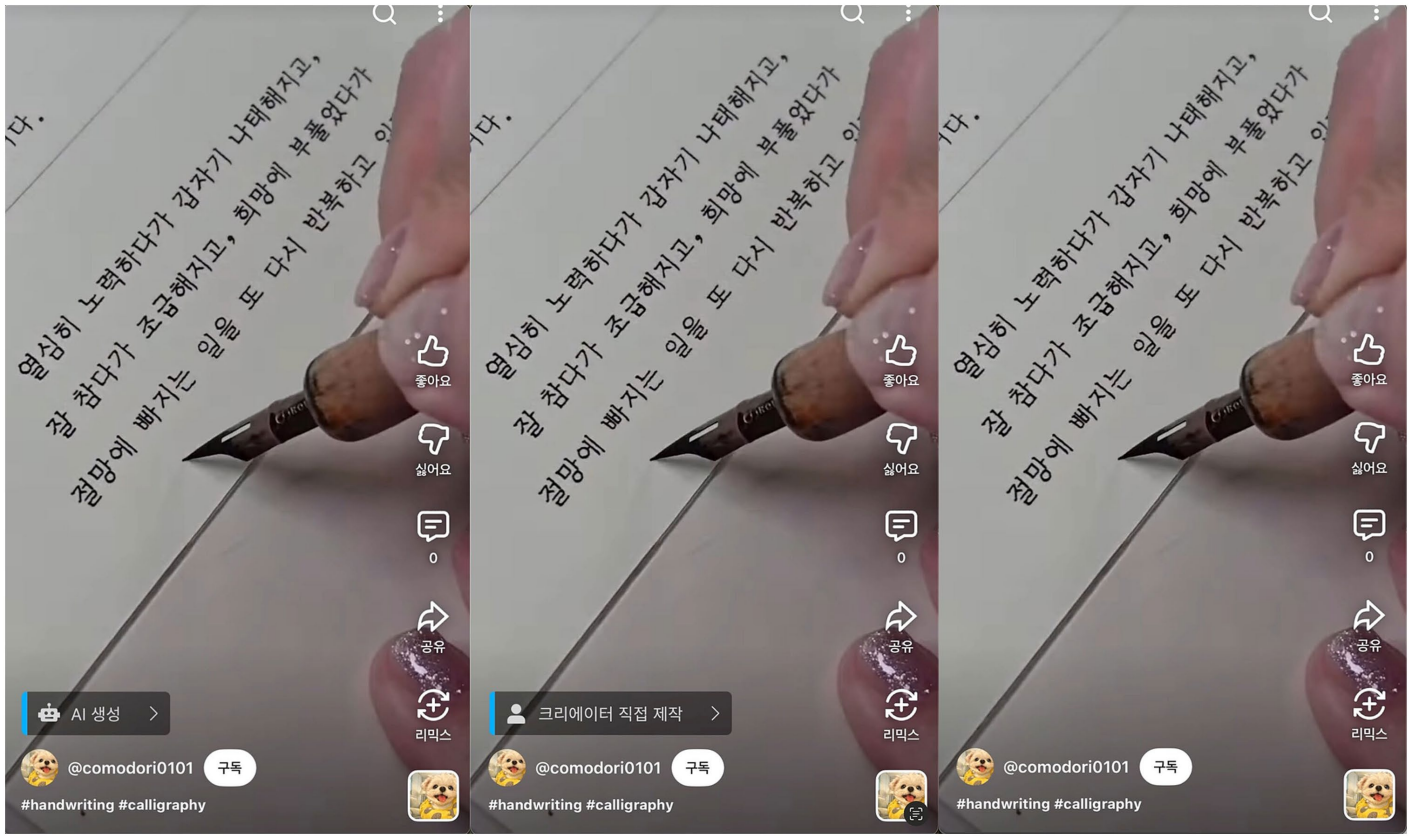
Example stimulus design for the eudaimonic content condition. (Adapted with permission from an Instagram Reel featuring a handwritten Vincent van Gogh quote by @beautiful_flower_write, posted to Instagram on November 2, 2025, and used in this article by permission of the copyright holder).

To avoid semantic interference from the lyrics, the video was also selected for background music, an instrumental piano composition (“Gymnopédie No. 1” by Erik Satie). This enabled participants to focus on the affective tone of the visual stimulus rather than verbal cues. An example of the eudaimonic stimulus is shown in Figure 2.

###### 4.3.3.1.2 Hedonic condition

**Figure 3:**
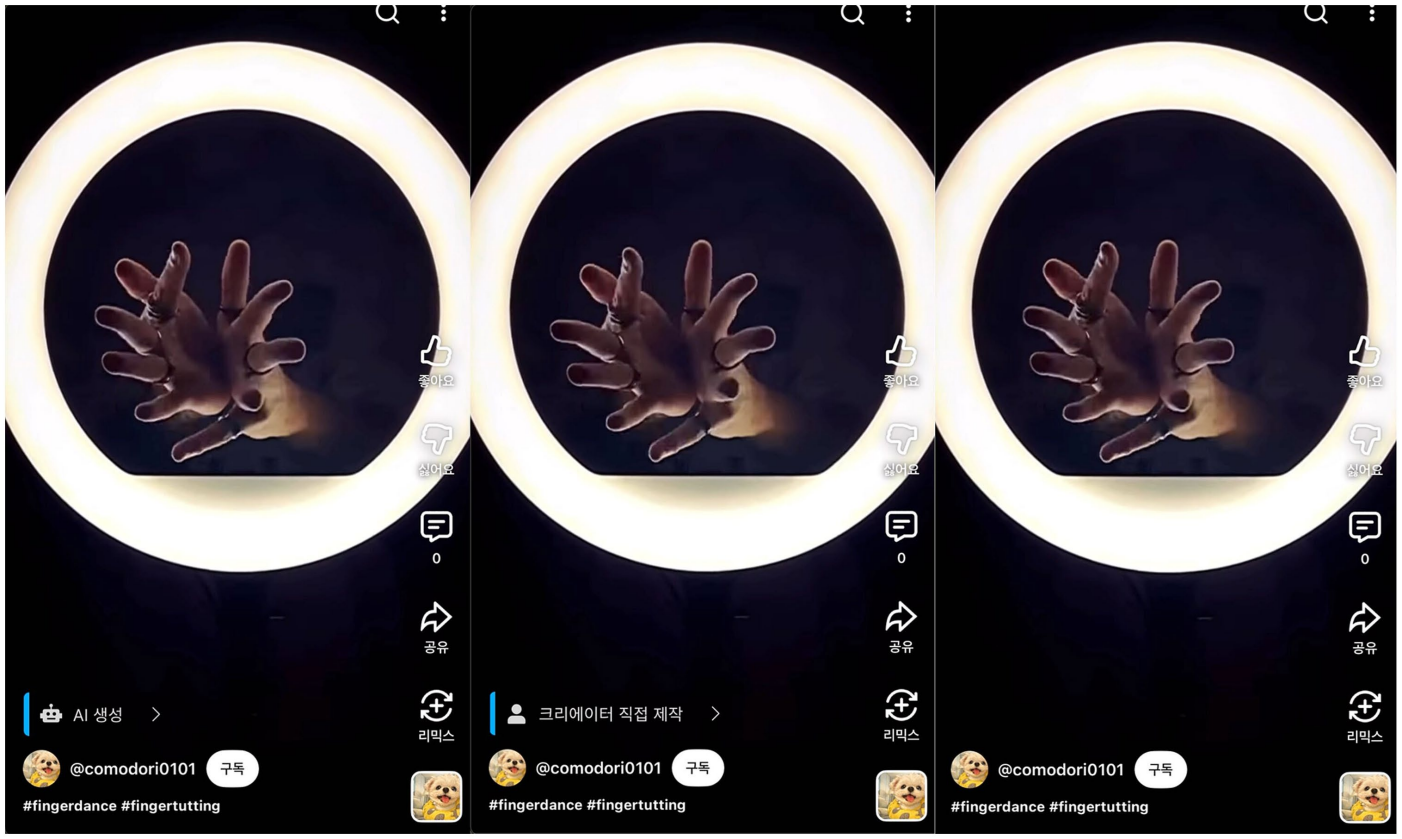
Example stimulus design for the hedonic content condition. (Adapted with permission from an Instagram Reel featuring a finger-tutting performance by @kamon__7, posted to Instagram on March 3, 2023, and used in this article by permission of the copyright holder).

In the hedonic condition, the stimulus was an approximately 12-s finger-tutting video in which the performer produced rapid geometric hand movements synchronized with a fast beat. The video emphasized physical skill, speed, and rhythmic precision without narrative elements or visual effects, thereby maximizing immediate sensory pleasure and aligning with established definitions of hedonic content (Oliver and Raney, 2011). The original instrumental electronic track (“Ltlp” by edIT) was retained to preserve audiovisual congruence and ecological validity. Although the musical styles differed from the eudaimonic condition, both stimuli exclusively used instrumental music, minimizing interpretive bias from lyrics or song familiarity while ensuring that differences in affective experience achieved the intended content-type manipulation. An example of the hedonic stimulus is presented in Figure 3.

##### 4.3.3.2 Factor B: label type

This study focused on how provenance labels are interpreted as value signals. Accordingly, the label presentation was designed to balance ecological validity with visual salience. Labels were displayed as semitransparent boxed overlays modeled on the standard interface of YouTube Shorts, thereby approximating real-world short-form platform environments.

To enhance visual conspicuity in line with emerging platform and regulatory norms that emphasize prominent AI disclosure, intuitive emojis were placed before the label text. All labels were presented in Korean using semantically equivalent expressions for widely used global platform terminology to ensure intuitive comprehension. In the “human-made” condition, the label “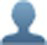 Creator Made” (literally, “directly made by the creator”) was used. The combination of the term (“made”) and a human-shaped icon was intended to signal human agency, physical performance, and intentional creative intervention. In contrast, the AI-generated condition displayed the label “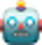 AI Generated,” with the term (“generated”) and a robot icon emphasizing automated production through algorithmic systems. This contrast was designed to differentiate human agency from machine-driven output and activate distinct attributions regarding effort and labor.

### 4.4 Measurement

All key variables were measured using established scales adapted for the algorithmic environment of short-form platforms and the context of GenAI. All items employed seven-point Likert-type scales (1 = strongly disagree, 7 = strongly agree). The full list of measurement items is reported in Table 1. Manipulation check items assessing label recognition and perceived content type were administered immediately after stimulus exposure. All analyses were controlled for label credibility, gender, age, and average short-form platform usage time. Label credibility was measured using an adapted scale from Flanagin and Metzger (2000) to account for individual differences in trust toward platform-provided labels.

**Table 1:** Measurement items and sources.

Dimension	Item	Content	References
PE	PE1	Creating this video must have required a substantial amount of time.	Kruger et al. (2004), Bellaiche et al. (2023), and Magni et al. (2024)
	PE2	The creator likely invested a great deal of energy in producing this video.	
	PE3	To perform this action or scene, the creator must have engaged in extensive practice or preparation behind the scenes.	
	PE4	This video does not appear to have been made casually; it seems to reflect careful and deliberate effort made during the production process.	
	PE5	The creator must have devoted considerable intellectual effort to developing the theme of this video and determining an original mode of expression.	
ACE	ACE1	I can adjust my feed so that content types I prefer are recommended more frequently through my reactions (e.g., likes).	Dogruel et al. (2022) and Hong (2022)
	ACE2	By engaging in behaviors such as liking or saving content, I can influence what appears in my recommended feed.	
	ACE3	By responding to this content, I can signal to the algorithm my preferred mode of content creation (human-made vs. AI-generated).	
	ACE4	I can manage my feed in the direction I prefer over time (e.g., toward human-created content).	
SCI	SCI1	I would check additional information (e.g., profiles, descriptions, or “more” sections) to find out how this content was created.	Khan (2017)
	SCI2	I would actively search for videos with a similar style or theme.	
	SCI3	I would intentionally consider following or subscribing so that the algorithm can better learn my preferences.	
	SCI4	I would like this video to signal to the algorithm that I want more content of this kind to be recommended in the future.	
	SCI5	I would use saving functions (e.g., collections, bookmarks) to record this video as part of my preference data.	
AL	AL1	I know that recommended videos on short-form platforms are based on my past viewing history and interaction data.	Gran et al. (2021) and Zarouali et al. (2021)
	AL2	I am aware that when I like or watch a video until the end, the algorithm uses this as information about my preferences.	
	AL3	I understand that users are shown different personalized recommendation feeds on short-form platforms.	
	AL4	I know that even when videos I do not want to see are recommended, they may be partly related to my previous usage behavior.	
PPP	PPP1	Recently, videos that appear to be generated by AI have frequently appeared in my feed.	Yang et al. (2024) and Jakesch et al. (2019)
	PPP2	Some of the recommended videos feel mechanically produced or have unclear origins.	
	PPP3	I feel that it is becoming increasingly difficult to find human-created content on short-form platforms.	
	PPP4	I often find it difficult to distinguish whether the videos I am watching are authentic or manipulated.	
	PPP5	I feel fatigued when browsing my feed due to the mass production of AI-generated content.	

PE was measured using five items adapted from Magni et al. (2024), drawing on prior conceptualizations of effort attribution (Kruger et al., 2004; Bellaiche et al., 2023), and tailored to the short-form video context. Based on Dogruel et al. (2022) and Hong (2022) and informed by Bandura’s (2006) contextualized efficacy framework, ACE was measured with four items assessing perceived feed adjustability, signaling effectiveness, and the capacity to intervene in algorithmic processes.

The scale for measuring SCI was adapted from Khan (2017) and refined to distinguish strategic intervention from passive engagement by embedding explicit algorithm-oriented purposes. Before responding to the SCI items, participants were instructed to consider familiar engagement behaviors, such as liking, saving, or searching for similar content, as intentional signals for shaping future video recommendations (i.e., “The following questions ask about behaviors you might use intentionally to influence what content your short-form video platform recommends to you in the future”). Five items assessed intentions related to signal transmission, learning induction, and information exploration. To measure PPP, a five-item scale was adapted based on prior work on algorithmic fatigue (Yang et al., 2024) and provenance uncertainty (Jakesch et al., 2019), capturing perceptions of AI content saturation and source ambiguity. Four items adapted from Gran et al. (2021) and Zarouali et al. (2021) assessed subjective knowledge of data collection, behavioral causality, and feed personalization, emphasizing perceived understanding rather than technical expertise in AL.

### 4.5 Data analysis

All analyses were conducted using SPSS 31.0 and PROCESS Macro 5.0 (Hayes, 2022), with the significance level set at *α* = 0.05. Consistent with the research model, analyses proceeded in two sequential phases. Phase 1 examined whether content provenance labeling influenced PE and whether this effect varied across contextual conditions (H1–H3). To test H1, an analysis of covariance (ANCOVA) was used to compare PE across label conditions, controlling for relevant demographic variables and label credibility. To test the moderating roles of content type and PPP (H2–H3), simple moderation analyses were conducted using PROCESS Macro Model 1.

Phase 2 investigated the psychological mechanisms through which PE affected SCI and the conditions under which this process operated (H4–H6). Serial mediation analyses using PROCESS Macro Model 6 were conducted to test the indirect pathway from PE to SCI via ACE, as well as the direct pathway from PE to SCI. Indirect effects were evaluated using bootstrapping with 5,000 resamples and 95% confidence intervals. Finally, moderated mediation analyses using PROCESS Macro Model 14 were conducted to test whether AL moderated the relationship between ACE and SCI (H6).

## 5 Results

### 5.1 Manipulation checks and preliminary analyses

#### 5.1.1 Sample characteristics

An online experimental survey was conducted with adult users who reported prior experience with short-form video platforms. After excluding responses with excessively short completion times or straightlining response patterns, 618 valid cases were retained for analysis. The sample was balanced by gender (50.3% men, 49.5% women), with age evenly distributed across cohorts, which included decade-based groups from the 20s to 50s and older (Table 2). Participants primarily reported moderate to high levels of short-form platform use, with the largest proportion viewing content for one to 2 h per day on average over the past week. Most respondents held a bachelor’s degree, and office or technical workers constituted the largest occupational group. Household income was commonly in the midrange category (2–4 million KRW per month). The experimental assignment was well balanced across label types (“human-made” vs. “AI-generated” vs. unlabeled) and content type (eudaimonic vs. hedonic) conditions, with no meaningful cell imbalance observed.

**Table 2:** Participants’ demographic characteristics (*N* = 618).

Category	Item	Frequency (*n*)	Percentage (%)
Gender	Male	311	50.3
	Female	306	49.5
	Other	1	0.2
Age	20–29	157	25.4
	30–39	155	25.1
	40–49	152	24.6
	50+	154	24.9
Education level	High school or below	67	10.8
	Currently enrolled in a university	60	9.7
	Bachelor’s degree	430	69.6
	Master’s degree	50	8.1
	Doctoral degree	11	1.8
Occupation	Student	67	10.8
	Office/technical worker	284	46.0
	Professional	50	8.1
	Self-employed/Freelancer	89	14.4
	Full-time homemaker	63	10.2
	Other (e.g., unemployed)	65	10.5
Monthly income	<KRW 2,000,000	46	7.4
	KRW 2,000,000–3,999,999	189	30.6
	KRW 4,000,000–5,999,999	172	27.8
	KRW 6,000,000–7,999,999	114	18.4
	KRW 8,000,000+	97	15.7
Total short-form video viewtime (past 7 days)	<10 min	28	4.5
	10–29 min	93	15.0
	30–59 min	145	23.5
	1–2 h	155	25.1
	2–3 h	97	15.7
	3 h+	100	16.2

#### 5.1.2 Reliability and validity of measures

Construct validity was assessed using exploratory factor analysis with principal component analysis and Varimax rotation. The data were suitable for factor analysis, as indicated by a high Kaiser–Meyer–Olkin (KMO) measure (0.911) and a significant Bartlett’s test of sphericity (*χ*^2^ = 12,282.63, df = 325, *p* < 0.001). Six factors were extracted based on an eigenvalue of greater than one criterion and scree plot inspection, accounting for 76.36% of the total variance. All items loaded onto their theoretically expected factors, with standardized loadings ranging from 0.721 to 0.872 and minimal cross-loadings (<0.30), supporting convergent and discriminant validity. Reliability analyses indicated a strong internal consistency across all constructs, with Cronbach’s α coefficients ranging from 0.868 to 0.929. The detailed factor loadings, explained variance, and reliability statistics are reported in Table 3.

**Table 3:** Validity and reliability of measurement scales.

Factor	Item	Factor loading	Eigenvalue	Variance explained (%)	Cronbach’s *ɑ*
SCI	SCI5	0.855	9.242	15.120	0.922
	SCI4	0.845			
	SCI2	0.799			
	SCI3	0.791			
	SCI1	0.766			
PE	PE4	0.872	3.907	14.694	0.920
	PE3	0.837			
	PE2	0.835			
	PE5	0.755			
	PE1	0.736			
PPP	PPP3	0.811	2.082	12.943	0.868
	PPP4	0.809			
	PPP5	0.776			
	PPP2	0.747			
	PPP1	0.723			
AL	AL2	0.849	1.950	11.840	0.894
	AL3	0.835			
	AL1	0.820			
	AL4	0.721			
ACE	ACE2	0.834	1.468	11.726	0.895
	ACE1	0.827			
	ACE3	0.797			
	ACE4	0.766			
LC	LC3	0.865	1.205	10.039	0.929
	LC2	0.860			
	LC1	0.840			
Total variance explained	76.361%				

1. Factor extraction was conducted using principal component analysis with Varimax rotation.

2. All factor loadings exceeded 0.50, and cross loadings were suppressed below 0.30.

3. Sampling adequacy was satisfactory (KMO = 0.911), and Bartlett’s test of sphericity was significant [*χ*^2^(325) = 12,282.634, *p* < 0.001].

4. PE, perceived effort; ACE, algorithmic curation efficacy; SCI, strategic curation intention; AL, algorithmic literacy; PPP, perceived platform pollution; LC, label credibility.

#### 5.1.3 Correlation analysis

Descriptive statistics and Pearson correlation analyses were conducted to examine the distributions and linear relationships among the key variables and assess potential multicollinearity (Table 4). AL and PPP showed relatively higher mean levels, whereas SCI exhibited the lowest mean, indicating a comparatively lower baseline propensity for strategic intervention. Correlation analyses revealed significant positive associations among all major variables (*p* < 0.01). The strongest correlation was observed between PE and SCI (*r* = 0.541), followed by the association between AL and ACE (*r* = 0.513). All correlation coefficients were below the conventional multicollinearity threshold (|r| < 0.80), suggesting that multicollinearity was not a concern for subsequent regression and PROCESS analyses.

**Table 4:** Correlations and descriptive statistics of key variables.

Dimension	1	2	3	4	5	6
1. SCI	1					
2. PE	0.541***	1				
3. ACE	0.372***	0.313***	1			
4. AL	0.189***	0.332***	0.513***	1		
5. PPP	0.237***	0.271***	0.386***	0.468***	1	
6. LC	0.510***	0.476***	0.335***	0.268***	0.132**	1
Mean	3.39	4.02	4.53	5.01	4.87	3.65
SD	1.48	1.40	1.26	1.23	1.20	1.38

1. ^***^p < 0.001, ^**^p < 0.01 (two-tailed).

2. All correlations are Pearson product–moment correlation coefficients.

3. Missing values were handled using listwise deletion.

4. PE, perceived effort; ACE, algorithmic curation efficacy; SCI, strategic curation intention; AL, algorithmic literacy; PPP, perceived platform pollution; LC, label credibility.

#### 5.1.4 Manipulation checks

Manipulation checks were conducted to confirm whether label and content types were perceived as intended. All participants correctly recognized the label information to which they were exposed, yielding a significant chi-square result (*χ*^2^ = 1236.00, *p* < 0.001). This confirmed the successful manipulation of the label type.

Content type manipulation was also effective. A large majority of the participants correctly classified the video they viewed as either eudaimonic or hedonic, with classification accuracy significantly exceeding chance levels [*χ*^2^(1) = 374.67, *p* < 0.001]. These results indicate that both experimental manipulations were clearly perceived and understood by participants.

### 5.2 Phase 1: value signaling and boundary conditions

Phase 1 examined whether content provenance labeling was translated into PE as a form of value attribution (H1) and whether this effect varied as a function of content type (H2) and PPP (H3).

#### 5.2.1 Main effect of label type on perceived effort (H1)

The assumption of homogeneity of variances was met in the ANCOVA [*F*(2, 615) = 1.36, *p* = 0.259]. The analysis revealed a significant main effect of label type on PE [*F*(2, 611) = 34.74, *p* < 0.001, *η*_ₚ_^2^ = 0.102]. Among the covariates, label credibility (*F* = 188.69, *p* < 0.001) and gender (*F* = 8.27, *p* = 0.004) were significant, whereas age and short-form usage time were not (Table 5).

**Table 5:** ANCOVA results for perceived effort by label type.

Source/variable	*N*	Adjusted mean (SE)	*F*-value	Significance	Partial eta squared	*Post hoc* comparison
Label type			34.74***	<0.001	0.102	Human-made ≈ unlabeled > AI-generated
Unlabeled	205	4.19 (0.082)				
Human-made	204	4.40 (0.082)				
AI-generated	209	3.48 (0.081)				
Covariates						
Gender			8.27**	0.004	0.013	
Age			1.30	0.255	0.002	
Usage			0.00	0.973	0.000	
LC			188.69***	<0.001	0.236	

1. The reference category for label type was “unlabeled”.

2. Values represent estimated marginal means, with covariates evaluated at their mean values.

3. *Post hoc* comparisons were conducted using Bonferroni correction.

4. **p < 0.01, ***p < 0.001.

5. LC, label credibility.

**Figure 4:**
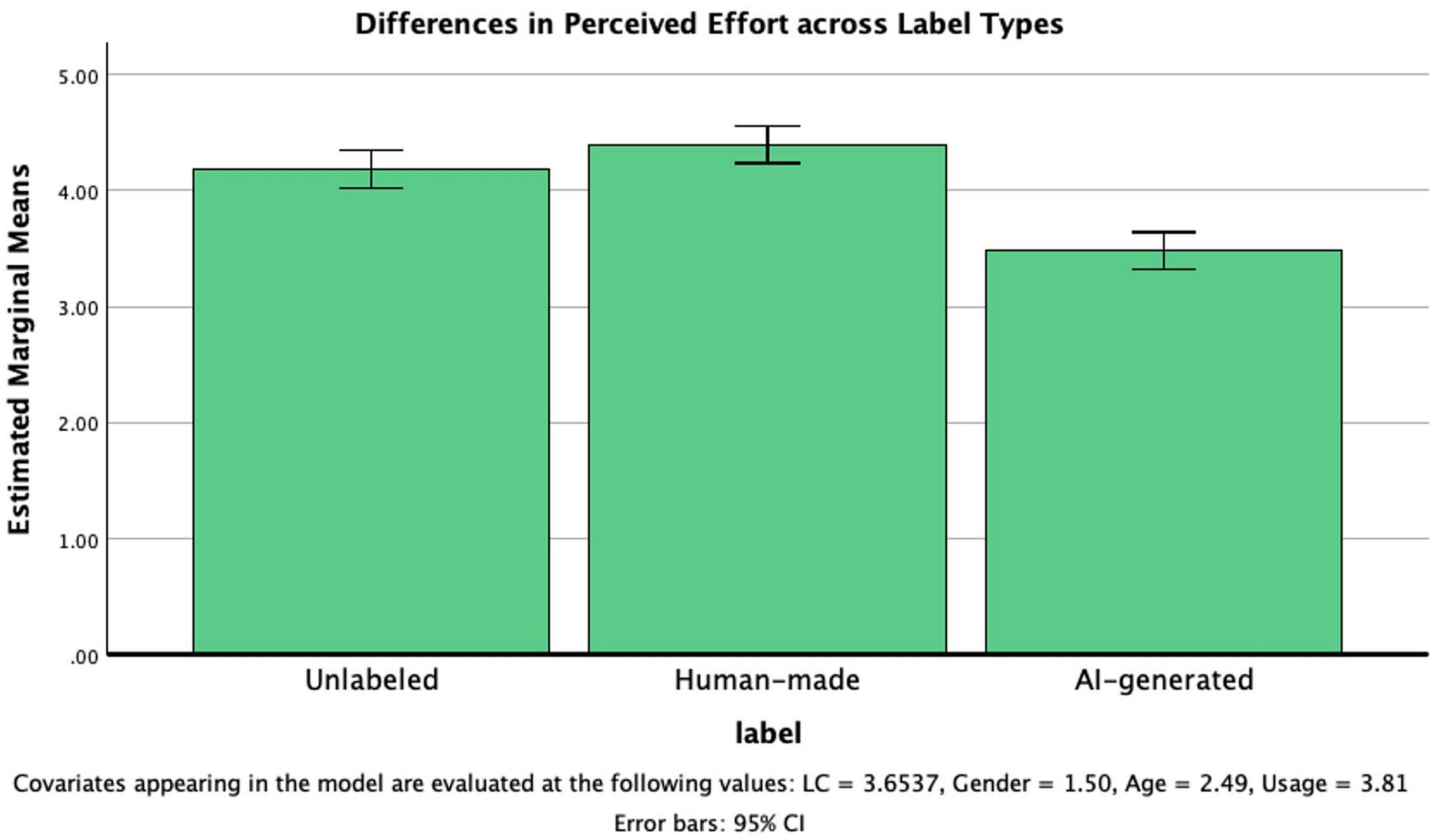
Differences in perceived effort across label types.

Bonferroni-adjusted *post hoc* comparisons showed that the “AI-generated” label condition (*M*_adj = 3.48) yielded significantly lower PE than the unlabeled condition (*M*_adj = 4.19). In contrast, the difference between the “human-made” label condition (*M*_adj = 4.40) and the unlabeled condition was not statistically significant (Δ*M* = 0.210, *p* = 0.213). Consistent with the predicted asymmetric pattern, only the AI-generated label significantly altered PE relative to the unlabeled baseline, whereas the human-made label did not differ from the baseline. H1 was supported (Figure 4).

Given the strong effect of label credibility, a moderation analysis (PROCESS Model 1) was conducted. The interaction between label type and label credibility was not significant (*p* = 0.474), and the interaction term did not explain additional variance (Δ*R*^2^ = 0.001). This indicates that label credibility affected the overall level of PE but did not alter relative differences between label types.

#### 5.2.2 Moderating effect of content type (H2)

The interaction between label and content types was not statistically significant [Δ*R*^2^ = 0.004, *F*(2, 608) = 1.96, *p* = 0.142]. Neither the interaction involving the “human-made” label (*B* = −0.240, *p* = 0.299) nor the interaction involving the “AI-generated” label (*B* = 0.214, *p* = 0.351) reached statistical significance, indicating that effort attribution did not differ systematically by content type. Although descriptive statistics suggested a larger numerical gap between the “human-made” and “AI-generated” labels under eudaimonic content than hedonic content, this difference was not statistically reliable. Importantly, the main effect of the “AI-generated” label remained strongly negative and significant (*B* = −0.706, *p* < 0.001), while no detectable moderation by content type was observed. Accordingly, H2 was not supported (Table 6).

**Table 6:** Results of the moderating effect of content type.

Predictor	Coefficient (*B*)	Standard error	*t*-Value	Significance
Intercept	1.982***	0.260	7.62	< 0.001
Control variables				
LC	0.470***	0.034	13.68	< 0.001
Gender	0.270**	0.093	2.90	0.004
Age	0.047	0.042	1.12	0.263
Usage	−0.010	0.034	−0.29	0.774
Independent variables				
“Human-made” label (X₁)	0.212	0.116	1.83	0.068
“AI-generated” label (X₂)	−0.706***	0.115	−6.16	< 0.001
Moderator				
Content type	0.196	0.163	1.20	0.230
Interaction terms				
Human-made label × content type	−0.240	0.231	−1.04	0.299
AI-generated label × content type	0.214	0.229	0.93	0.351

1. The reference category for label type was “unlabeled.” Content type was coded as 0 = eudaimonic and 1 = hedonic.

2. *p < 0.05, **p < 0.01, ***p < 0.001.

3. Model fit: *R*-squared = 0.324, *F*(9, 608) = 32.36, *p* < 0.001.

4. Interaction effect: change in *R*-squared = 0.004, *F*(2, 608) = 1.96, *p* = 0.142.

5. LC, label credibility.

#### 5.2.3 Moderating the role of perceived platform pollution (H3)

The interaction between label type and PPP was not statistically significant, and the incremental explanatory power of the model was negligible [Δ*R*^2^ = 0.002, *F*(2, 608) = 0.79, *p* = 0.453]. Neither the interaction involving the “human-made” label (*B* = 0.009, *p* = 0.922) nor the interaction involving the “AI-generated” label (*B* = −0.099, *p* = 0.280) reached statistical significance, indicating that PPP did not moderate labeling effects on PE. Although descriptive patterns suggested slightly larger differences between the “human-made” and “AI-generated” label conditions at higher levels of PPP, these tendencies were not statistically reliable.

Importantly, the negative main effect of the “AI-generated” label on PE remained strong and statistically significant in the model (*B* = −0.689, *p* < 0.001), while no detectable moderation by PPP was observed. In contrast, the “human-made” label did not produce a significant premium relative to the unlabeled condition, even under high PPP (*B* = 0.187, *p* = 0.101). Accordingly, H3 was not supported (Table 7).

**Table 7:** Results of the moderating effect of perceived platform pollution.

Predictor	Coefficient (B)	Standard error	*t*-value	Significance
Intercept	2.191***	0.259	8.46	<0.001
Control variables				
LC	0.448***	0.034	13.21	<0.001
Gender	0.197*	0.092	2.13	0.034
Age	0.045	0.042	1.07	0.284
Usage	−0.013	0.033	−0.40	0.687
Independent variables				
“Human-made” label	0.187	0.114	1.64	0.101
“AI-generated” label	−0.689***	0.113	−6.12	<0.001
Moderator				
PPP	0.244***	0.062	3.94	<0.001
Interaction terms				
“Human-made” label × PPP	0.009	0.093	0.10	0.922
“AI-generated” label × PPP	−0.099	0.092	−1.08	0.280

1. The reference category for label type was “unlabeled”.

2. *p < 0.05, **p < 0.01, ***p < 0.001.

3. Model fit: *R*-squared = 0.349, *F*(9, 608) = 36.27, *p* < 0.001.

4. Interaction effect: change in *R*-squared = 0.002, *F*(2, 608) = 0.79, *p* = 0.453.

5. LC, label credibility; PPP, perceived platform pollution.

### 5.3 Phase 2: behavioral mechanisms

Phase 2 examined the psychological mechanisms through which value perceptions formed by labeling (operationalized as PE) were translated into SCI.

#### 5.3.1 Mediation through perceived effort and algorithmic curation efficacy (H4–5)

To test the dual-pathway mechanism linking label type to SCI via PE and ACE, a serial multiple mediation analysis was conducted using PROCESS Macro Model 6, controlling for gender, age, short-form usage time, and label credibility (Table 8). Results showed that PE had a positive significant effect on ACE (*B* = 0.176, SE = 0.039, *t* = 4.50, *p* < 0.001), supporting H4a. ACE positively predicted SCI (*B* = 0.219, SE = 0.041, *t* = 5.33, *p* < 0.001), supporting H5. In addition to this indirect pathway, PE exerted a strong positive direct effect on SCI even after controlling for ACE (*B* = 0.391, SE = 0.040, *t* = 9.70, *p* < 0.001), supporting H4b.

**Table 8:** Results of the serial multiple mediation analysis.

Variable	Model 1: PE	Model 2: ACE	Model 3: SCI
	Coefficient (SE)	Coefficient (SE)	Coefficient (SE)
Intercept	1.939 (0.261)***	2.651 (0.263)***	−0.007 (0.288)
Control variables			
LC	0.473 (0.034)***	0.227 (0.038)***	0.290 (0.040)***
Gender	0.269 (0.093)**	0.416 (0.091)***	−0.221 (0.094)*
Age	0.048 (0.043)	−0.191 (0.041)***	0.043 (0.042)
Usage time	−0.001 (0.034)	0.046 (0.033)	−0.006 (0.033)
Independent variables			
“Human-made” label	0.210 (0.116)	−0.002 (0.113)	−0.041 (0.114)
“AI-generated” label	−0.703 (0.115)***	0.066 (0.115)	0.111 (0.116)
Mediators			
PE		0.176 (0.039)***	0.391 (0.040)***
ACE			0.219 (0.041)***
Model summary	*R*-squared = 0.315; *F* = 46.83***	*R*-squared = 0.206; *F* = 22.66***	*R*-squared = 0.407; *F* = 52.15***

1. The reference category for label type was “unlabeled”.

2. *p < 0.05, **p < 0.01, ***p < 0.001.

3. PE, perceived effort; ACE, algorithmic curation efficacy; SCI, strategic curation intention.

Bootstrapping analyses (5,000 resamples) revealed asymmetric indirect effects across label types (Table 9). Relative to the unlabeled condition, the “AI-generated” label significantly reduced PE (*B* = −0.703, *p* < 0.001), producing negative indirect effects through both pathways. The AI label yielded a significant negative indirect effect via the direct normative pathway [AI → PE → SCI; effect = −0.275, 95% CI (−0.392, −0.166)] and a significant negative serial indirect effect via the rational pathway [AI → PE → ACE → SCI; effect = −0.027, 95% CI (−0.049, −0.010)]. Importantly, these effects were not equivalent in magnitude. The direct PE–SCI pathway was substantially stronger, whereas the efficacy-mediated pathway operated as a smaller but statistically significant additional mechanism.

**Table 9:** Results of indirect effects based on bootstrapping.

Path	Indirect effect	Bootstrap SE	95% confidence interval (lower, upper)
Simple mediation			
“Human-made” label → PE → SCI	0.082	0.045	[−0.001, 0.174]
“AI-generated label” → PE → SCI	−0.275	0.057	[−0.392, −0.166]
“Human-made” label → ACE → SCI	0.000	0.024	[−0.047, 0.050]
“AI-generated” label → ACE → SCI	0.015	0.027	[−0.038, 0.070]
Serial mediation			
“Human-made” label → PE → ACE → SCI	0.008	0.005	[0.000, 0.020]
“AI-generated” label → PE → ACE → SCI	−0.027	0.010	[−0.049, −0.010]

1. Indirect effects were estimated using bootstrapping with 5,000 resamples.

2. An indirect effect is considered statistically significant if the 95% confidence interval does not include zero.

3. PE, perceived effort; ACE, algorithmic curation efficacy; SCI, strategic curation intention.

**Figure 5:**
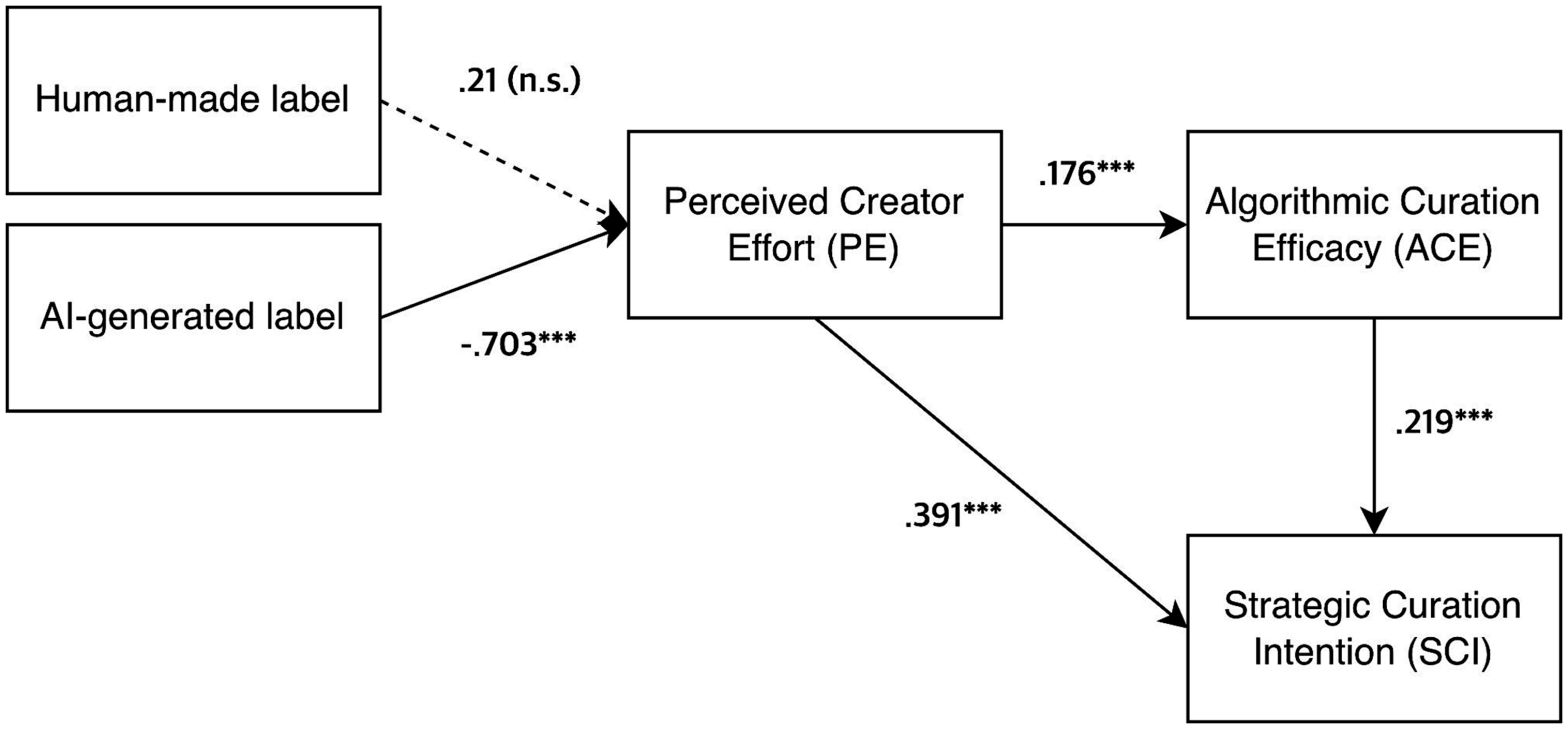
Path diagram of the serial multiple mediation model.

In contrast, the “human-made” label did not produce significant indirect effects relative to the unlabeled condition. Neither the simple indirect pathway [Human → PE → SCI; 95% CI (−0.001, 0.174)] nor the serial indirect pathway [Human → PE → ACE → SCI; effect = 0.008, 95% CI (0.000, 0.020)] reached statistical significance. These results support the proposed dual-pathway model (H4a, H4b, H5) while demonstrating that “AI-generated” labels systematically weaken normative and efficacy-based pathways, thereby reducing SCI (Figure 5).

#### 5.3.2 Moderated mediation by algorithmic literacy (H6)

A moderated mediation analysis using PROCESS Macro Model 14 was conducted to test whether algorithmic literacy (AL) moderated the relationship between ACE and SCI, with PE as the antecedent mediator. Gender, age, short-form usage time, label credibility, and label type were included as covariates (Table 10). The interaction between ACE and AL (ACE × AL) was not statistically significant (*B* = 0.026, SE = 0.024, *t* = 1.08, *p* = 0.282), and the increase in explained variance attributable to the interaction was negligible (Δ*R*^2^ = 0.001). Thus, no detectable moderation of the ACE–SCI relationship by AL was observed. H6 was therefore not supported.

**Table 10:** Results of the moderating effect of algorithmic awareness.

Predictor	Coefficient (*B*)	Standard error	*t*-Value	Significance
Intercept	0.836**	0.279	3.00	0.003
Control variables				
LC	0.296***	0.039	7.51	<0.001
Gender	−0.196*	0.093	−2.10	0.036
Age	0.031	0.042	0.72	0.469
Usage time	−0.010	0.033	−0.31	0.759
Label type	0.064	0.058	1.11	0.267
Independent variable				
PE	0.409***	0.040	10.29	<0.001
Mediator				
ACE	0.279***	0.045	6.27	<0.001
Moderator				
AL	−0.139**	0.045	−3.11	0.002
Interaction term				
ACE × AL	0.026	0.024	1.08	0.282

1. LC, label credibility; PE, perceived effort; AL, algorithmic literacy; ACE, algorithmic curation efficacy.

2. *p < 0.05, **p < 0.01, ***p < 0.001.

3. Model summary: *R*-squared = 0.417, *F*(9, 608) = 48.33, *p* < 0.001.

Despite the absence of moderation, the model showed significant main effects. Both ACE (*B* = 0.279, *p* < 0.001) and PE (*B* = 0.409, *p* < 0.001) exerted significant positive effects on SCI, whereas AL showed an unexpected negative association with SCI (*B* = −0.139, *p* = 0.002). Because AL was introduced primarily as a moderator, this negative main effect should be interpreted as exploratory. Bootstrapping analyses further indicated that the indirect effect of PE on SCI via ACE was significant at all levels of AL. The index of moderated mediation was not significant [Index = 0.004, 95% CI (−0.006, 0.016)], indicating that the mediation mechanism did not vary detectably by users’ AL (Table 11).

**Table 11:** Results of the moderated mediation analysis.

Index/condition	Effect	Bootstrap SE	95% confidence interval (lower, upper)
Index of moderated mediation	0.004	0.006	[−0.006, 0.016]
Conditional indirect effects			
Low AL (−1 SD)	0.043	0.016	[0.017, 0.078]
Mean AL	0.048	0.017	[0.021, 0.085]
High AL (+1 SD)	0.054	0.020	[0.020, 0.098]

1. AL, algorithmic literacy.

2. Effects were estimated using bootstrapping with 5,000 resamples.

3. An effect was considered statistically significant if the 95% confidence interval did not include zero.

## 6 Discussion

### 6.1 Algorithmic discounting and human default

The findings of this study indicate that the impact of content provenance labels on users’ value evaluations is fundamentally asymmetric. The “AI-generated” label produced a pronounced discounting effect by significantly reducing PE, whereas the “human-made” label did not generate a meaningful value premium relative to the unlabeled condition. This suggests that provenance labeling functions as a powerful evaluative cue primarily when it discloses algorithmic generation rather than when it affirms human authorship.

The negative effect of the AI label can be interpreted through expectancy violations theory. Users implicitly assume human creation as the normative standard for short-form content (Millet et al., 2023; Jia et al., 2024). Within this expectation structure, the AI-generated label operated as a deviant signal that reframed the content as a low-effort, automated output, thereby triggering users’ psychological resistance and value depreciation (Rae, 2024; Chen et al., 2025).

Meanwhile, the absence of a significant effect for the “human-made” label provided indirect evidence for the human default. For users, human authorship does not constitute novel or reward-worthy information but remains an assumed baseline. Additional analyses further demonstrated that although label credibility influenced the absolute level of PE, it did not moderate the relative differences between label conditions. The persistent non-significance between the “human-made” label and unlabeled conditions thus reflected entrenched default assumptions rather than skepticism toward labeling.

At the same time, the human default should be understood as contingent on both the current, transitional phase of GenAI adoption and the low-involvement context of short-form platforms. In environments characterized by immediacy and heuristic processing, unlabeled content is readily assimilated as human-made (Gamage et al., 2025). However, if AI-generated content were to surpass a critical threshold and dominate the ecosystem, a lack of labels could increasingly signal uncertainty, allowing explicit human authorship labels to acquire premium value through scarcity. Labeling effects were thus shown to operate through a dual structure: a clear penalty associated with AI disclosure and a default assumption of human creation. At this stage, provenance labeling functions primarily as a means of identifying algorithmic intrusion and enabling defensive differentiation in increasingly automated content environments.

### 6.2 The dual pathway mechanism of perceived effort

This study demonstrated that users’ SCIs are shaped through a dual-pathway psychological mechanism integrating rational judgment and normative reward motives. PE operates simultaneously through an indirect pathway, in which it enhances ACE and subsequently increases SCI (H4a, H5), and a direct pathway, in which it influences SCI independently of efficacy beliefs (H4b).

The indirect pathway reflects how higher perceived creator efforts strengthen users’ beliefs that their reactions can meaningfully influence recommendation systems. Users interpret curation as a form of strategic investment, grounded in the expectation that present engagement can shape future feed outcomes. Meanwhile, the direct pathway revealed the operation of the norm of reciprocity (Morales, 2005).

Strategic curation here emerges as a phenomenon in which efficacy-based control beliefs coexist with effort-based response tendencies. Crucially, this study demonstrated that “AI-generated” labels suppress both pathways simultaneously, but not symmetrically. The negative effect through the direct PE–SCI pathway was substantially larger than the serial indirect effect through the ACE-mediated pathway. This pattern suggests an asymmetric dual-path effect rather than an equally balanced dual-path penalty. By signaling an absence of human effort, the AI label primarily weakened the direct effort-based route to SCI, while also weakening perceived ACE by foregrounding machine-driven generation (Yang et al., 2024; Chen et al., 2025; Heimstad et al., 2025). As a result, the AI label became a demotivating cue that attenuated user engagement mainly through the direct PE–SCI route, with an additional but more limited effect through the efficacy-mediated route.

### 6.3 AI penalty and non-detected contextual moderation

The absence of statistically significant moderation effects across the examined conditions (e.g., content type, PPP) suggests that the AI-label penalty was not detectably conditioned by these contextual factors in the present sample (H2–3). Rather than providing definitive evidence of invariance across contexts, these findings should be interpreted more cautiously as consistent with the possibility that provenance labels operate as salient heuristic cues in low-involvement short-form environments. In this sense, users’ value judgments may be driven less by systematic content and context processing than by immediately actionable provenance cues.

The provenance label appeared to function as a salient attribution cue that was not detectably moderated by genre-based distinctions across content types. In the low-involvement consumption context of short-form platforms, the label generated a powerful first impression that categorically framed content as “machine-made,” independent of narrative depth or emotional resonance (Rae, 2024; Chen et al., 2025). From the perspective of the heuristic–systematic model, environments characterized by minimal cognitive investment privilege efficient cues over content-based evaluation, allowing provenance labels to structurally preempt systematic processing (Sundar, 2020; Chen et al., 2025).

A similar logic applies to PPP. Effort attribution proved more responsive to local, salient cues than to users’ global beliefs about the platform environment. The absence of detected moderation by PPP suggests that users’ effort attributions may have been more responsive to the local label cue than to broader perceptions of platform degradation. One possible interpretation is that the AI-generated label itself functioned as a sufficiently salient cue of algorithmic intrusion (Jakesch et al., 2019; Wittenberg et al., 2025). These findings are consistent with the interpretation that, in rapid, judgment-driven short-form environments, GenAI labels may function as salient attribution heuristics that signal reduced human effort.

### 6.4 Exploratory pattern of algorithmic literacy and strategic curation

The absence of a moderating effect for AL (H6), combined with its negative main effect, offers an exploratory insight into how user agency may operate in algorithmic environments. AL did not strengthen the translation of efficacy into behavior; instead, it showed an unexpected negative association with SCI. Because AL was introduced primarily as a moderator rather than as a focal explanatory variable, this pattern should be interpreted cautiously as exploratory evidence consistent with a possible literacy paradox. In this interpretation, increased understanding of system complexity may elicit fatigue and skepticism rather than empowerment (Yang et al., 2024). Users who more clearly recognize the scale and opacity of recommendation systems may perceive their individual actions as inconsequential, fostering algorithmic cynicism or resignation (DeVito et al., 2017; Gran et al., 2021; Swart, 2021). Greater technical knowledge may therefore intensify perceived loss of control rather than enhance it.

At the same time, ACE’s behavioral effect observed in earlier analyses indicates that it operates as an independent, central mechanism that is largely decoupled from technical knowledge. In short-form environments, user agency depends less on the factual understanding of algorithms than on the belief that meaningful intervention is possible. This distinction highlights the primacy of efficacy (belief) over knowledge (fact) in motivating strategic engagement. Accordingly, initiatives promoting AL should move beyond conveying system mechanics to designs that make the impact of user feedback experientially salient. This would cultivate efficacy beliefs rather than merely increasing technical comprehension.

## 7 Conclusion

### 7.1 Theoretical implications: users as algorithmic negotiators and the value of effort

This study reconceptualized media users as active negotiating agents who strategically calibrate their digital environments through behavioral signals. Actions such as “likes,” traditionally interpreted as expressions of immediate preference, here become intentional interventions through which users seek to shape algorithmic learning and personalize recommendation outcomes. The findings further demonstrate that PE, which is embedded in content production, constitutes a central driver of strategic behavior.

Effort attribution was found to be asymmetric: while the “human-made” label failed to generate a meaningful value premium relative to the unlabeled condition, the “AI-generated” label created a strong penalty that significantly reduced PE. This illustrated the widespread existing assumption of humans as the default creators (Pennycook et al., 2020; Wittenberg et al., 2025). This renders explicit “human-created” labels largely redundant under current conditions.

The study also found that PE structures behavioral translation through a dual-pathway mechanism. PE strengthens SCI indirectly by enhancing ACE while also exerting a direct effect on SCI that can be interpreted as consistent with reciprocity-based reward motivation (Morales, 2005; Buell and Norton, 2011). Importantly, these pathways were asymmetric in strength, with the direct PE–SCI pathway emerging as the dominant route and the ACE-mediated pathway functioning as a smaller supplementary mechanism. This finding suggests that AI-generated labels primarily suppress the direct translation of perceived effort into strategic curation intention, while also weakening efficacy-based strategic intervention. More broadly, the results indicate that understanding user agency in GenAI environments requires shifting attention away from attitude- or trust-centered accounts and toward effort attribution as a core analytical unit determining how agency is enacted.

Finally, this study has extended the literature on labor illusion by showing that its operation changes in GenAI–driven content environments. Prior research has demonstrated that visible human labor enhances value evaluations in physical product markets (Kruger et al., 2004; Buell and Norton, 2011). In short-form content contexts, however, visually observable performance is generally interpreted as human labor by default. Once an AI-generated label is introduced, the same performance is reattributed to algorithmic production, leading to a discount in perceived value. This suggests that the observed effect reflects a shift in how effort is attributed. Accordingly, in digital content ecosystems, the labor illusion depends less on the visibility of effort than on how effort attribution is structured and sustained.

### 7.2 Practical and policy implications: from heuristic warnings to systematic value certification

Platform labeling strategies have reached a critical juncture. They may need to move beyond one-dimensional information disclosure and recalibrate the nature of the signals that users receive. The current binary labeling regime generates stigmatizing effects toward AI-generated content while structurally undermining users’ algorithmic efficacy. The discounting effect triggered by the *AI-generated* label operated as a context-insensitive heuristic, prompting immediate rejection responses before substantive engagement with content. Notably, such rejection may represent a form of “defensive efficacy”—a proactive attempt to filter the environment—rather than mere loss of agency (Yang et al., 2024; Yuan et al., 2025). Future labeling should therefore distinguish between approach-oriented efficacy, which seeks to optimize content, and avoidance-oriented efficacy, which aims to protect users from unwanted algorithmic intrusion.

Accordingly, future labeling policies should aim to shift users from heuristic processing toward the systematic evaluation of creative effort. This requires expanding labeling from a focus on source to process, making visible the degree and nature of human involvement. Rather than warning-oriented disclosures, platforms could explore effort visualization interfaces that communicate human creative work and value through intuitive cues. In this framework, labels must evolve from indicating what tools were used to how much human effort was invested.

Such design changes must be aligned with broader ecosystem-preservation strategies. Although the “human-made” label did not yield a premium under current conditions due to the human default assumption, this outcome highlights the need for proactive intervention as AI-generated content proliferates. A possible policy direction would be a dual-track approach that combines AI risk regulation with the active promotion of human creative value.

Crucially, this promotion cannot rely on symbolic markers alone. Instead, it requires strategic feedback loops that allow users to experience how their engagement meaningfully supports human creators. For example, signals could indicate how specific actions increase the visibility or weighting of human-created content. Such mechanisms could restore users’ ACE and counter algorithmic resignation. Redefining labeling as a system of value certification rather than risk warning may provide a useful safeguard against the dystopian trajectory anticipated by the “Dead Internet Theory,” preserving both user agency and human creative value in increasingly automated media environments.

### 7.3 Limitations and directions for future research

Although this study provided an integrated account of user psychology in the era of GenAI by conceptualizing users as algorithmic negotiators and foregrounding effort attribution, several limitations should be noted. First, the analysis was confined to short-form video platforms. Given the immediacy and low-involvement nature of short-form content, effort evaluation may operate differently in more high-involvement domains (e.g., news, documentaries, long-form artistic productions). Future research should therefore extend this framework across a wider range of modalities and genres (e.g., text, images, music) to examine whether AI-induced PE discounting varies by content type.

Second, the ecological validity of the experimental stimuli warrants further consideration. To ensure causal clarity, this study relied on a binary distinction between “human-made” and “AI-generated” labels, whereas real-world production increasingly involves hybrid human–AI collaboration. Future work should disaggregate degrees of AI involvement to identify potential threshold effects in effort attribution. The study also controlled for personal cues and biases by excluding creators’ faces. In contexts where facial or vocal presence is more salient, “human-made” labels may function as stronger value signals, meriting further investigation.

Third, the sample was limited to users in South Korea. While this context is characterized by high AI adoption and intensive short-form consumption, cultural differences may have shaped how human creativity and automation were evaluated. Cross-cultural research is therefore needed to assess the generalizability of the human default assumption and AI penalty effects across diverse cultural settings and content genres.

In addition, smaller moderation effects may not have been detectable with the present sample structure. Future studies with larger samples should more precisely estimate boundary conditions for content type, PPP, and AL.

Finally, the study measured strategic curation using self-reported intentions rather than observed behavior. Although intention is a strong predictor of action, the intention–behavior gap cannot be fully ignored. Future research should employ platform log data and field experiments to examine whether SCI results in observable recommendation outcomes. Further studies may also test alternative labeling designs (e.g., effort visualization indicators) to evaluate their effectiveness in restoring users’ ACE.

## Data Availability

The raw data supporting the conclusions of this article will be made available by the authors, without undue reservation.
