# Supplementary material for: Human-made vs. AI-generated: how provenance labels drive strategic curation via perceived effort
Source: Front Psychol. 2026 Jun 3;17:1840483. doi: 10.3389/fpsyg.2026.1840483 (PMC13272402; doi:10.3389/fpsyg.2026.1840483)
Supplement: Supplementary file 1 [file Supplementary_File_1.docx]

Supplementary Material

## Supplementary Figures(experimental stimulus)


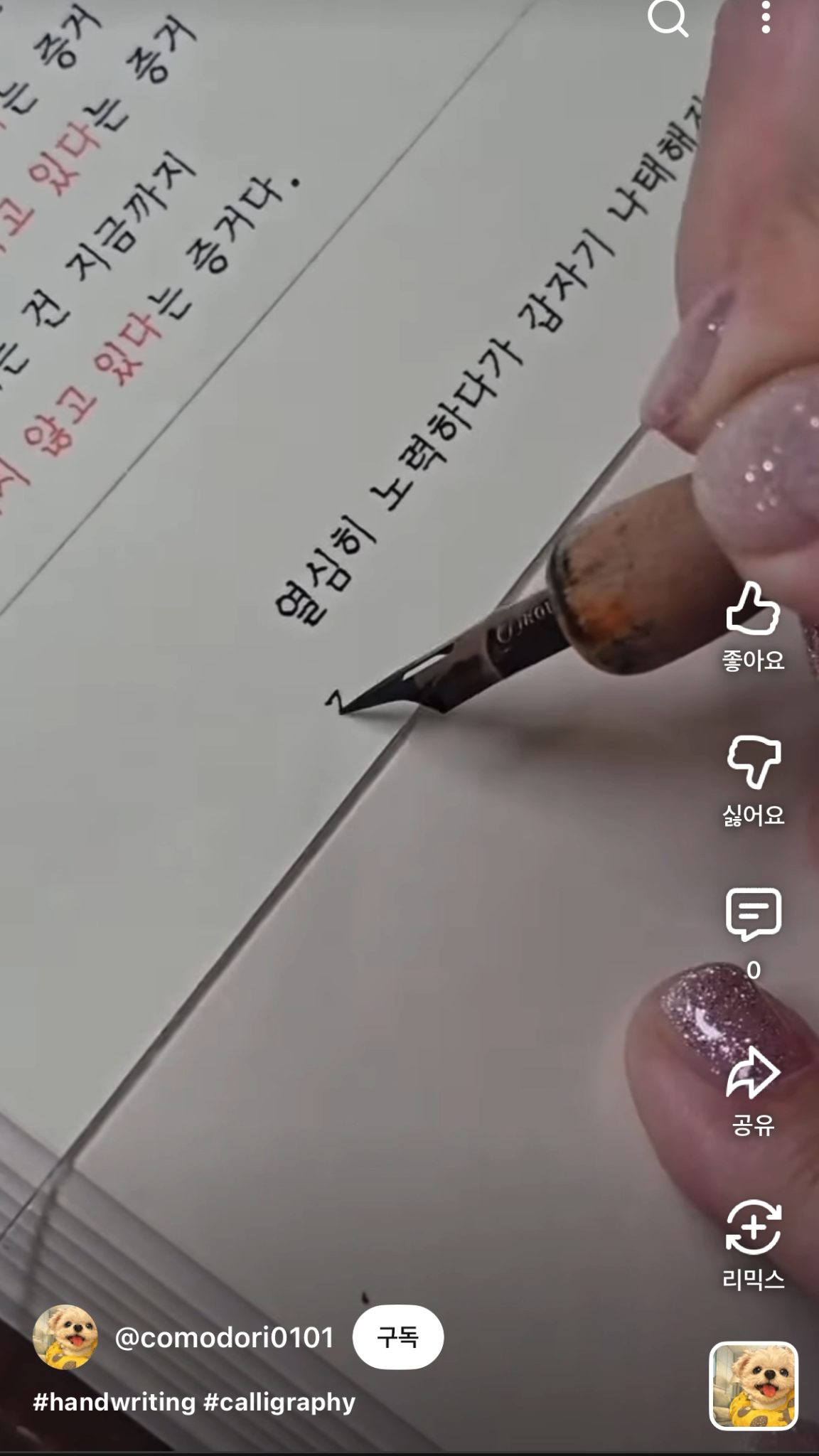

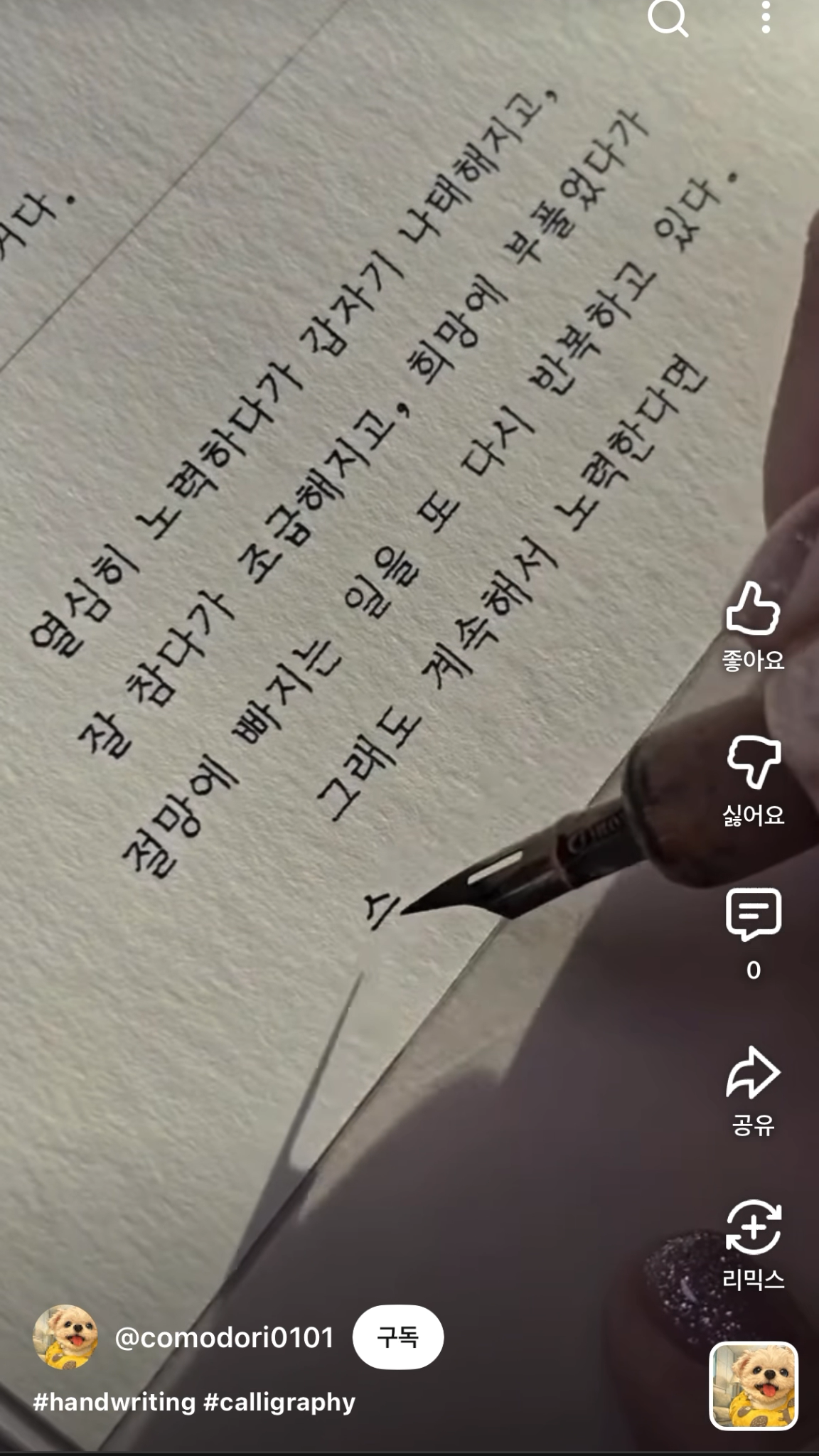

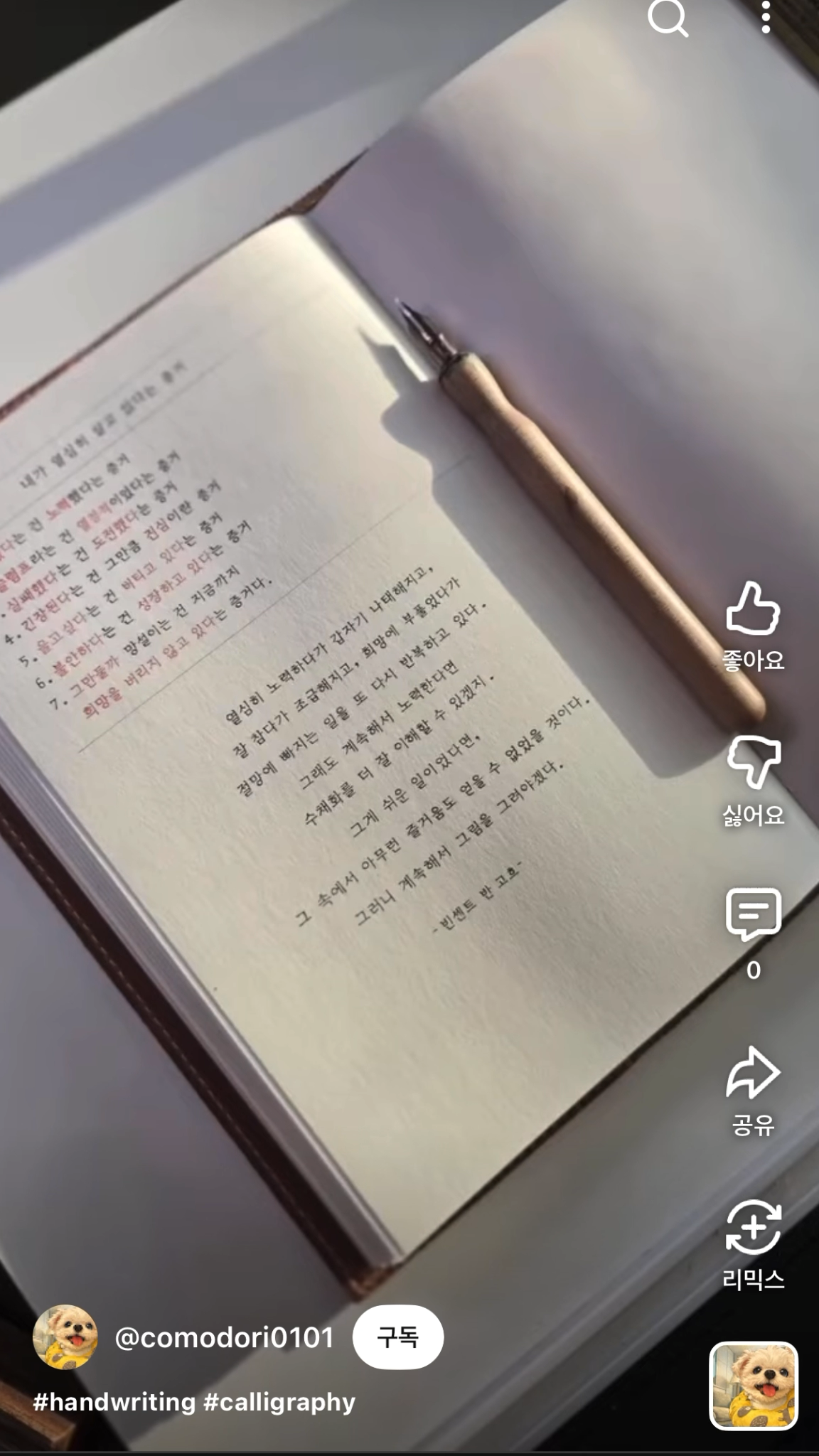


**Supplementary Figure 1.**
**No-label condition for the eudaimonic stimulus.** Screenshots from an Instagram Reel by @beautiful_flower_write, posted to Instagram on November 2, 2025. Showing the stimulus presented without a provenance label. Adapted with permission and used in this article by permission of the copyright holder.

**
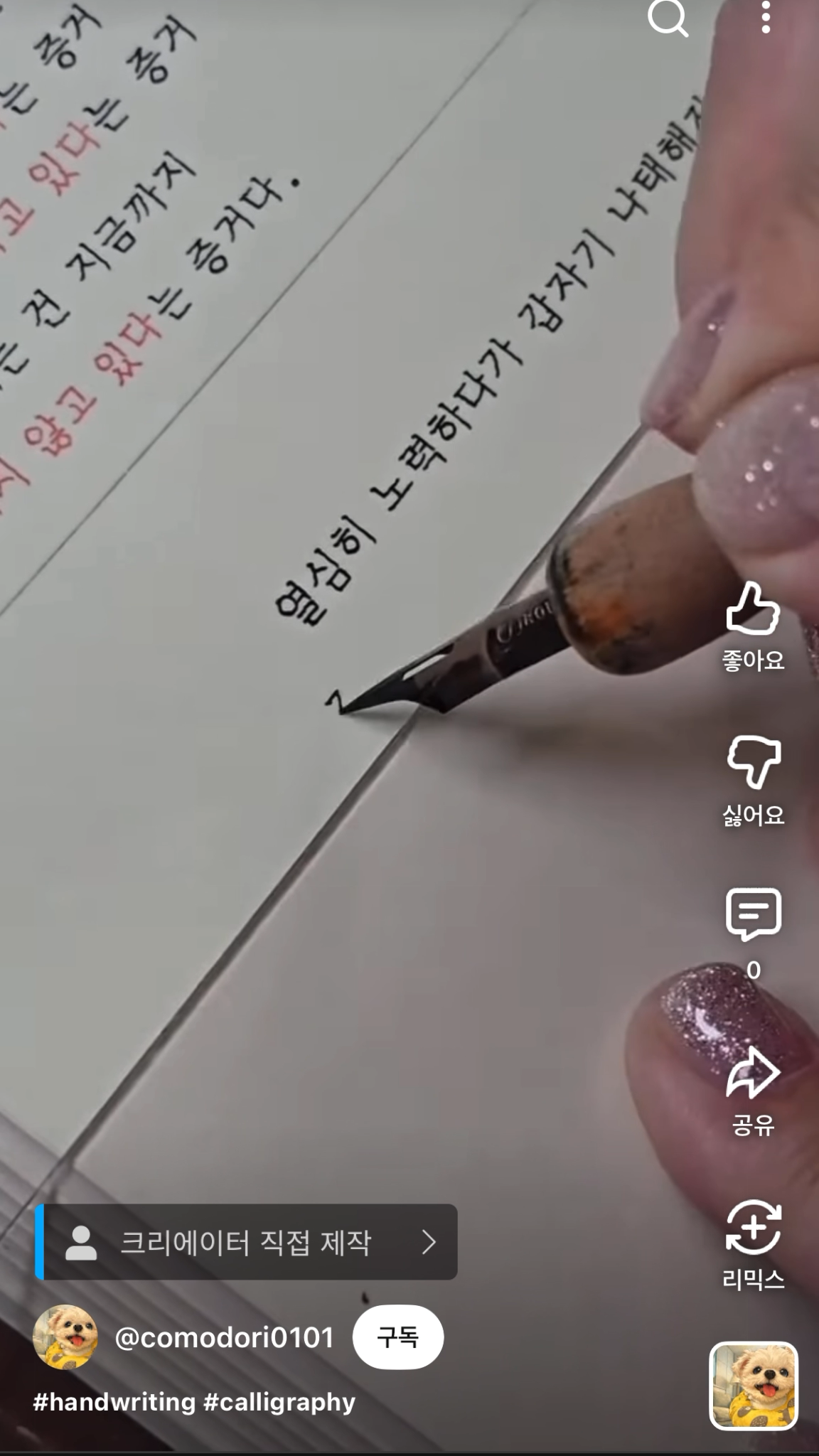
**
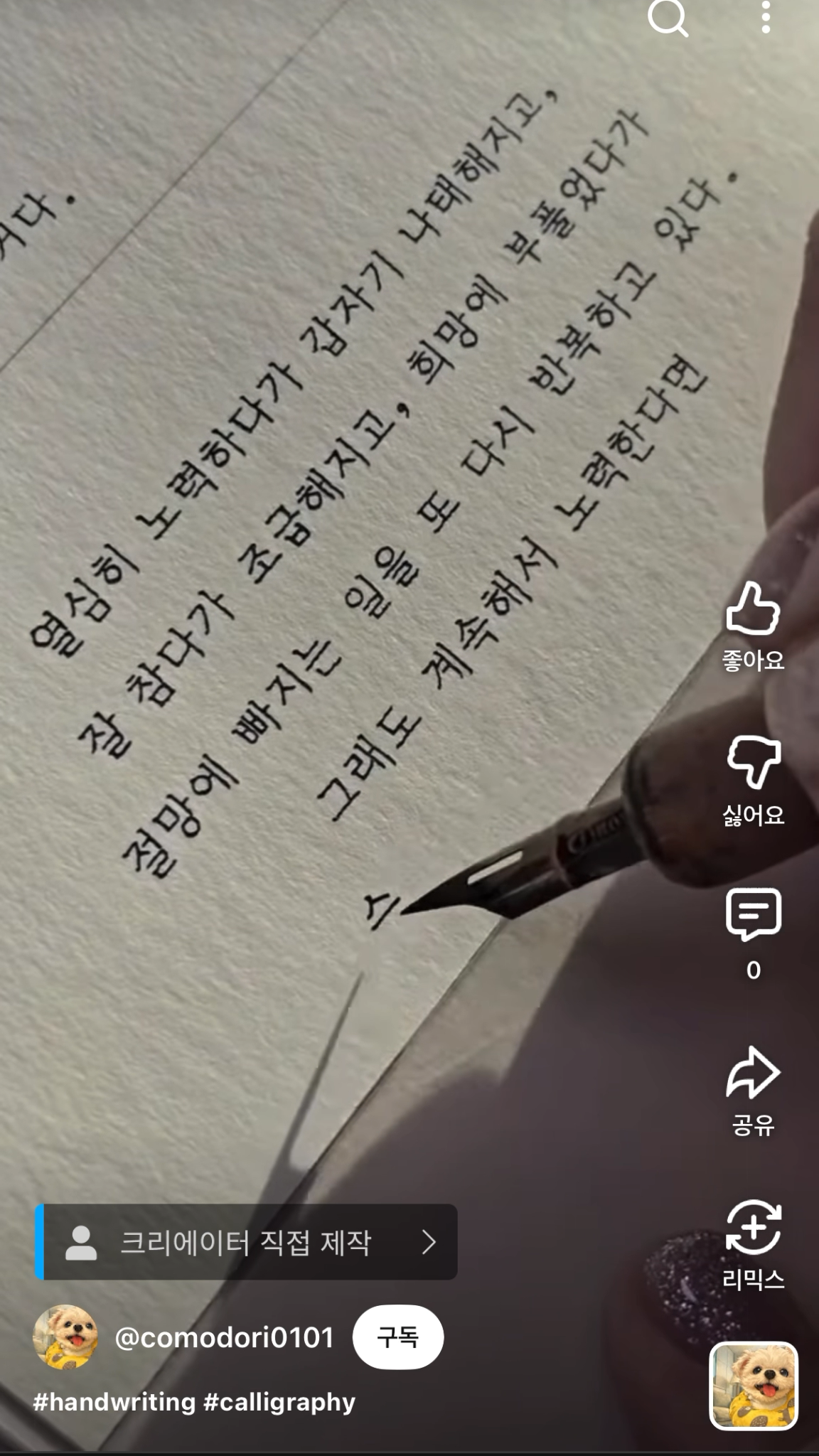

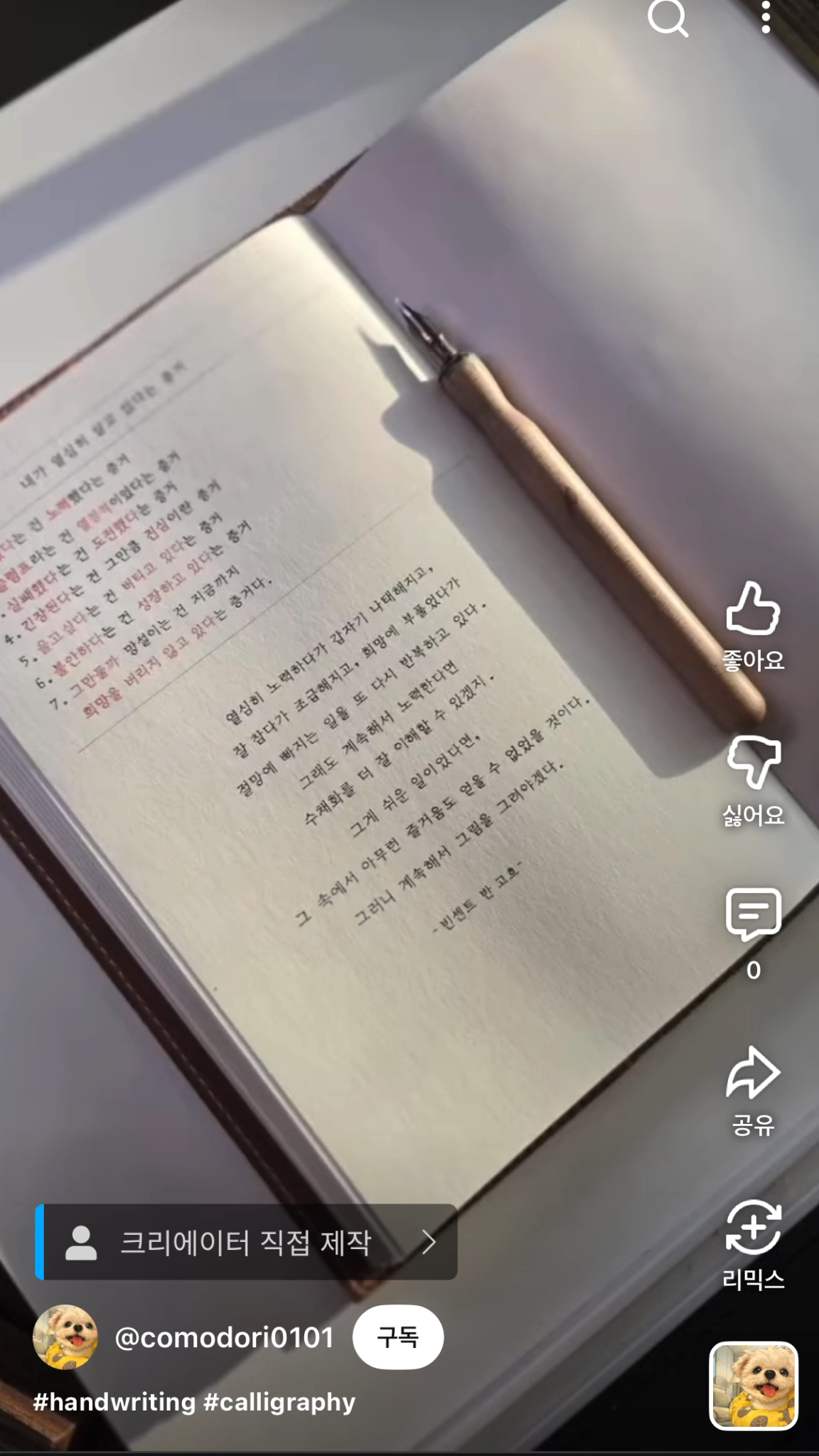


**Supplementary Figure 2.**
**Human-made label condition for the eudaimonic stimulus.** Screenshots from an Instagram Reel by @beautiful_flower_write, posted to Instagram on November 2, 2025, showing the stimulus presented with a Human-made provenance label. Adapted with permission and used in this article by permission of the copyright holder.

**
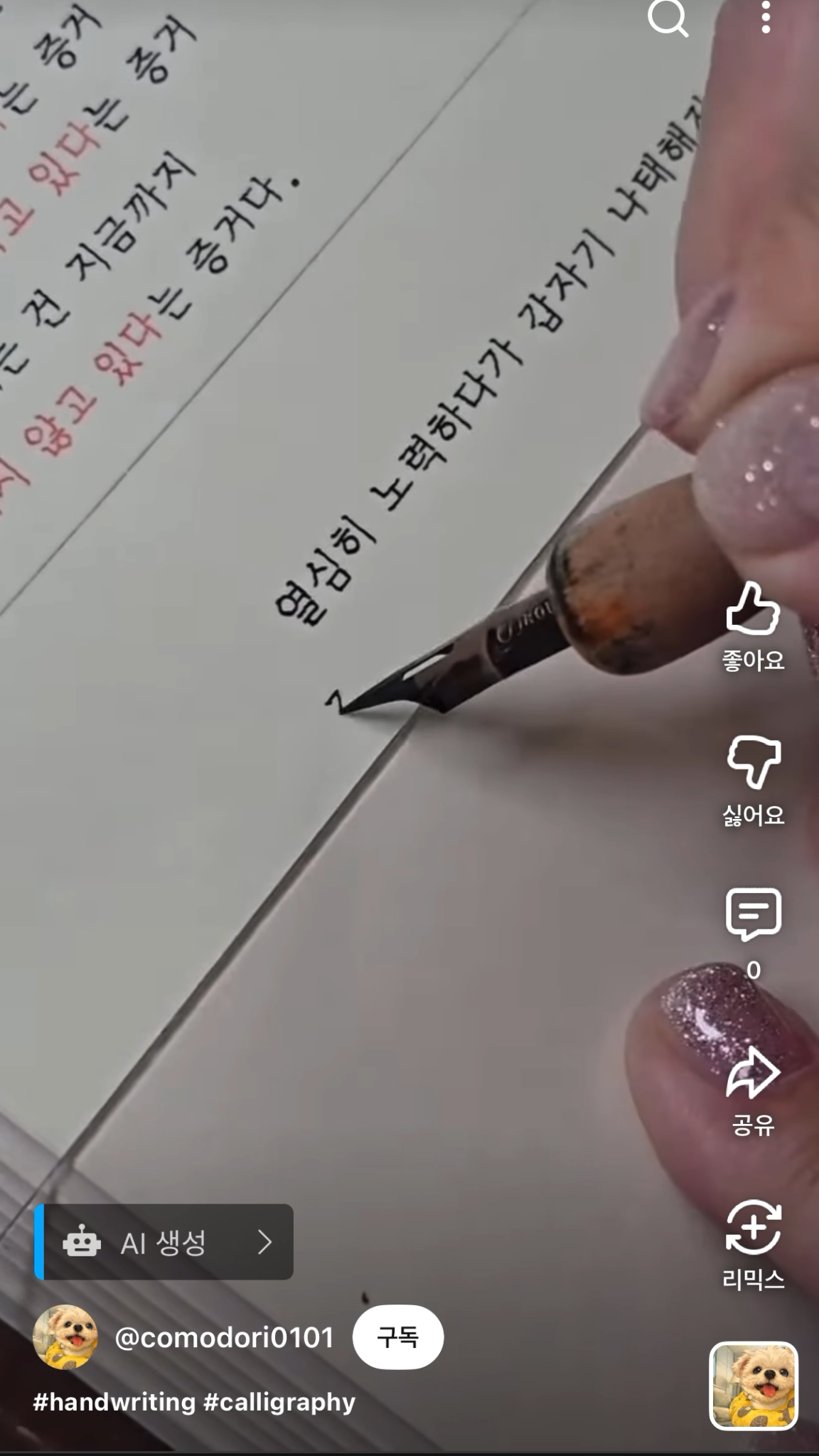
**
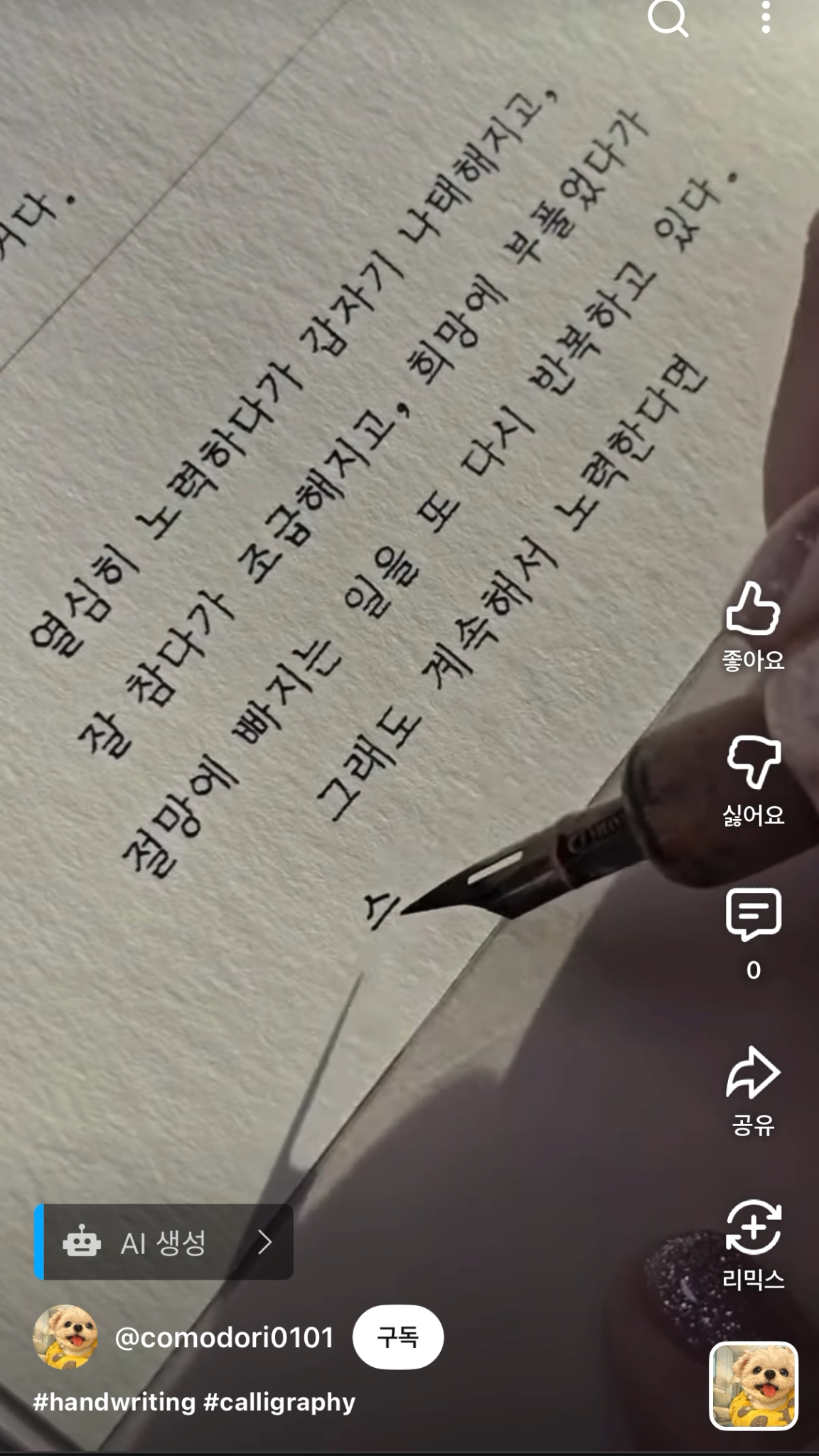

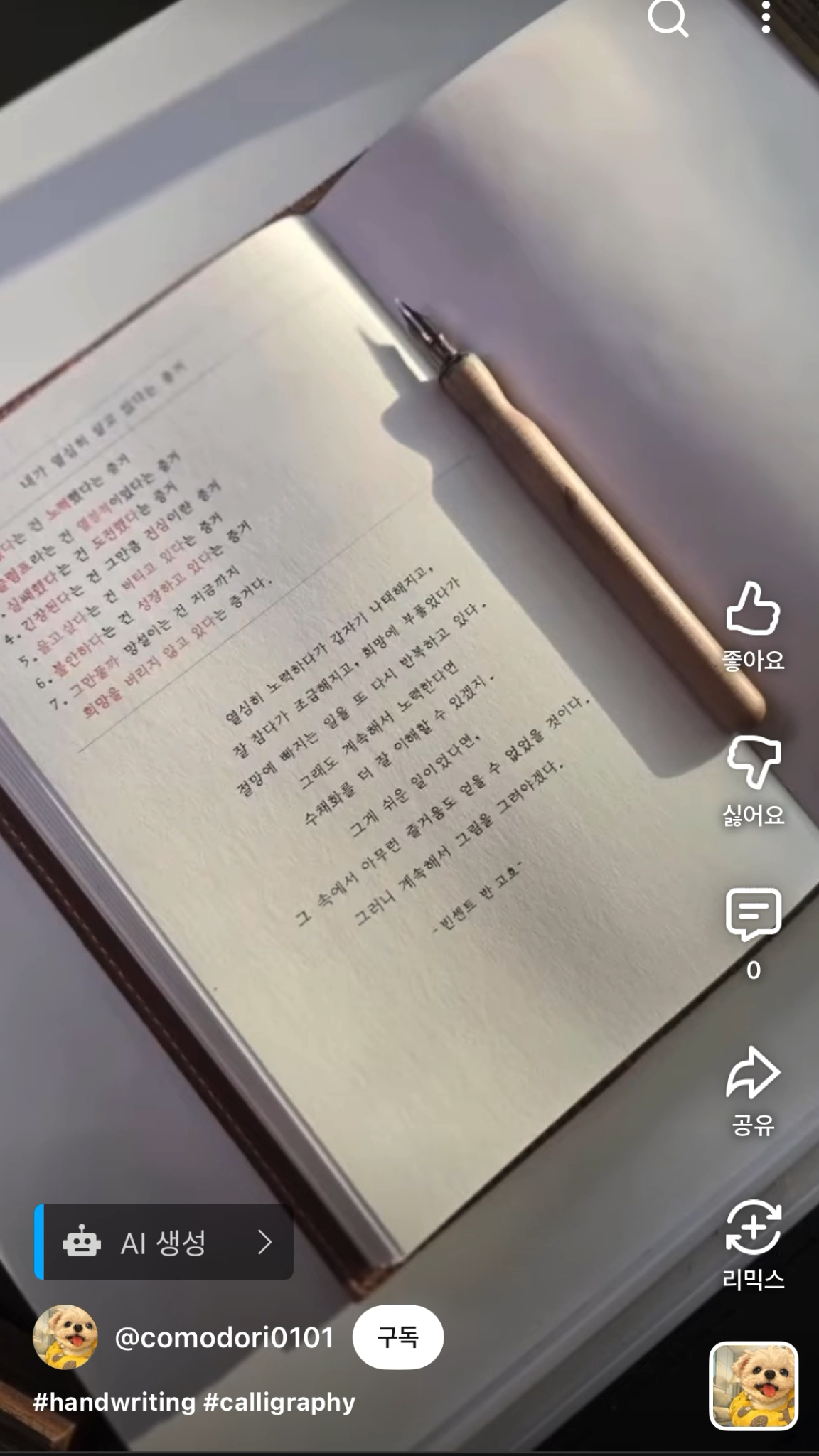


**Supplementary Figure 3.**
**AI-generated label condition for the eudaimonic stimulus.** Screenshots from an Instagram Reel by @beautiful_flower_write, posted to Instagram on November 2, 2025, showing the stimulus presented with an AI-generated provenance label. Adapted with permission and used in this article by permission of the copyright holder.


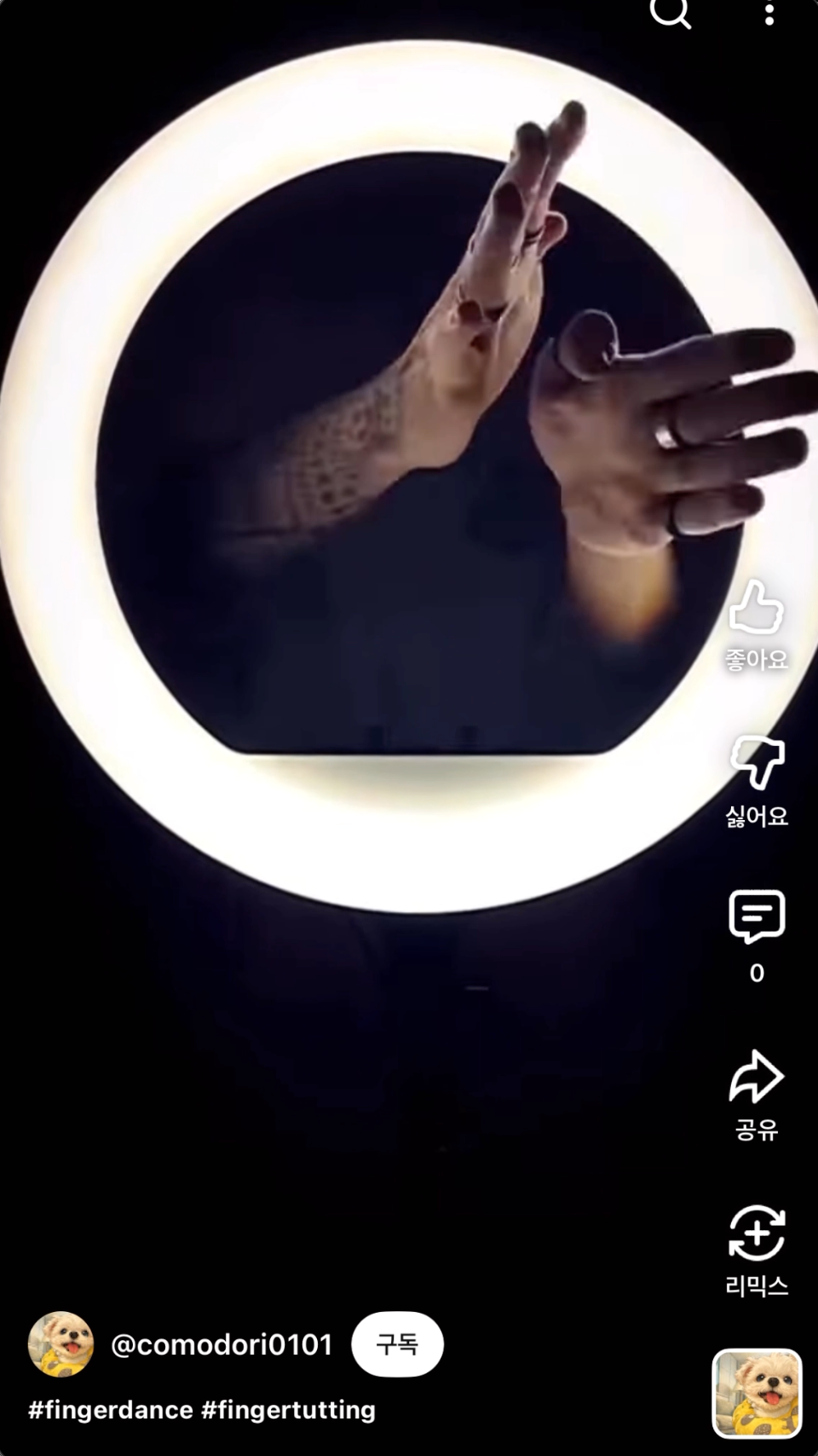

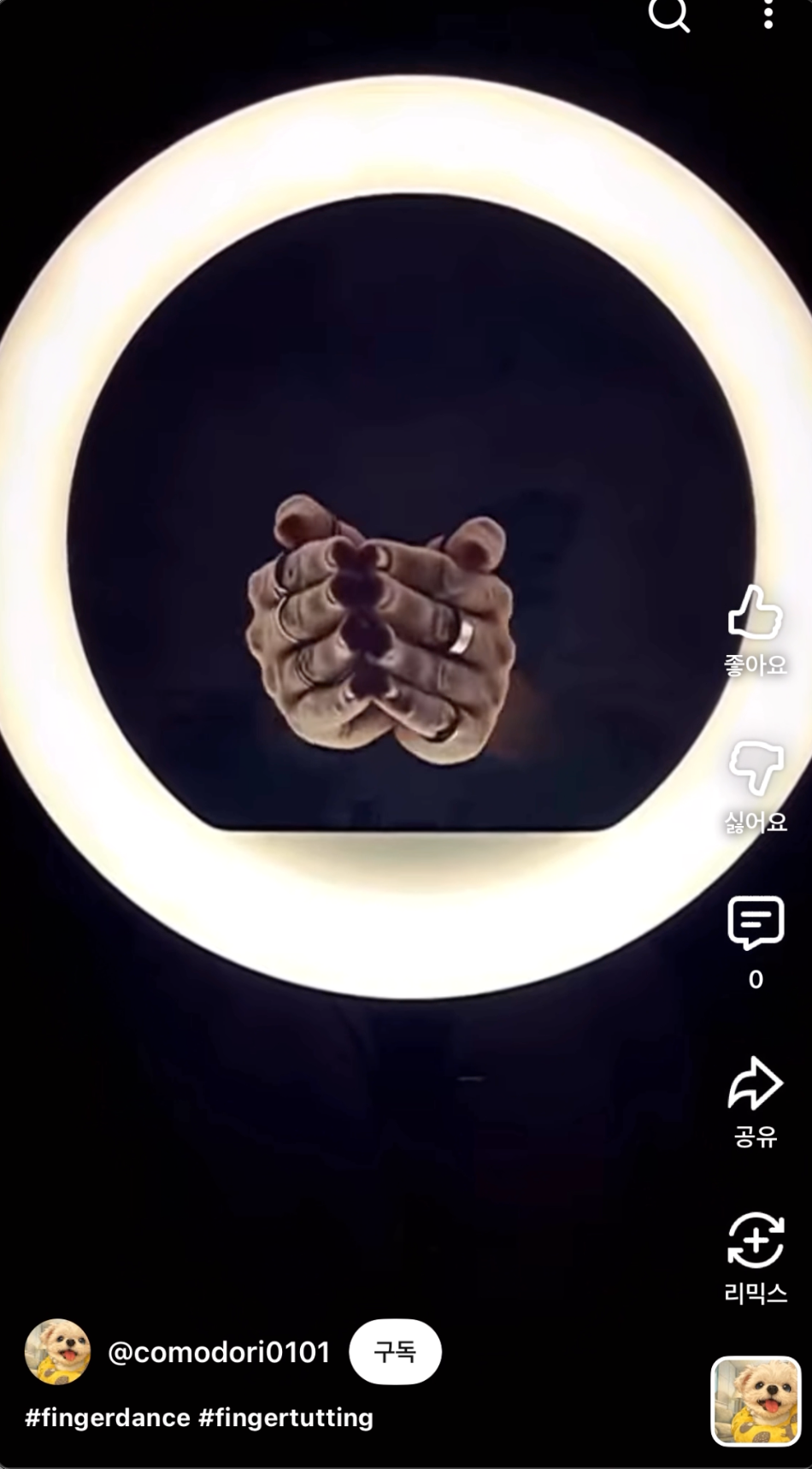

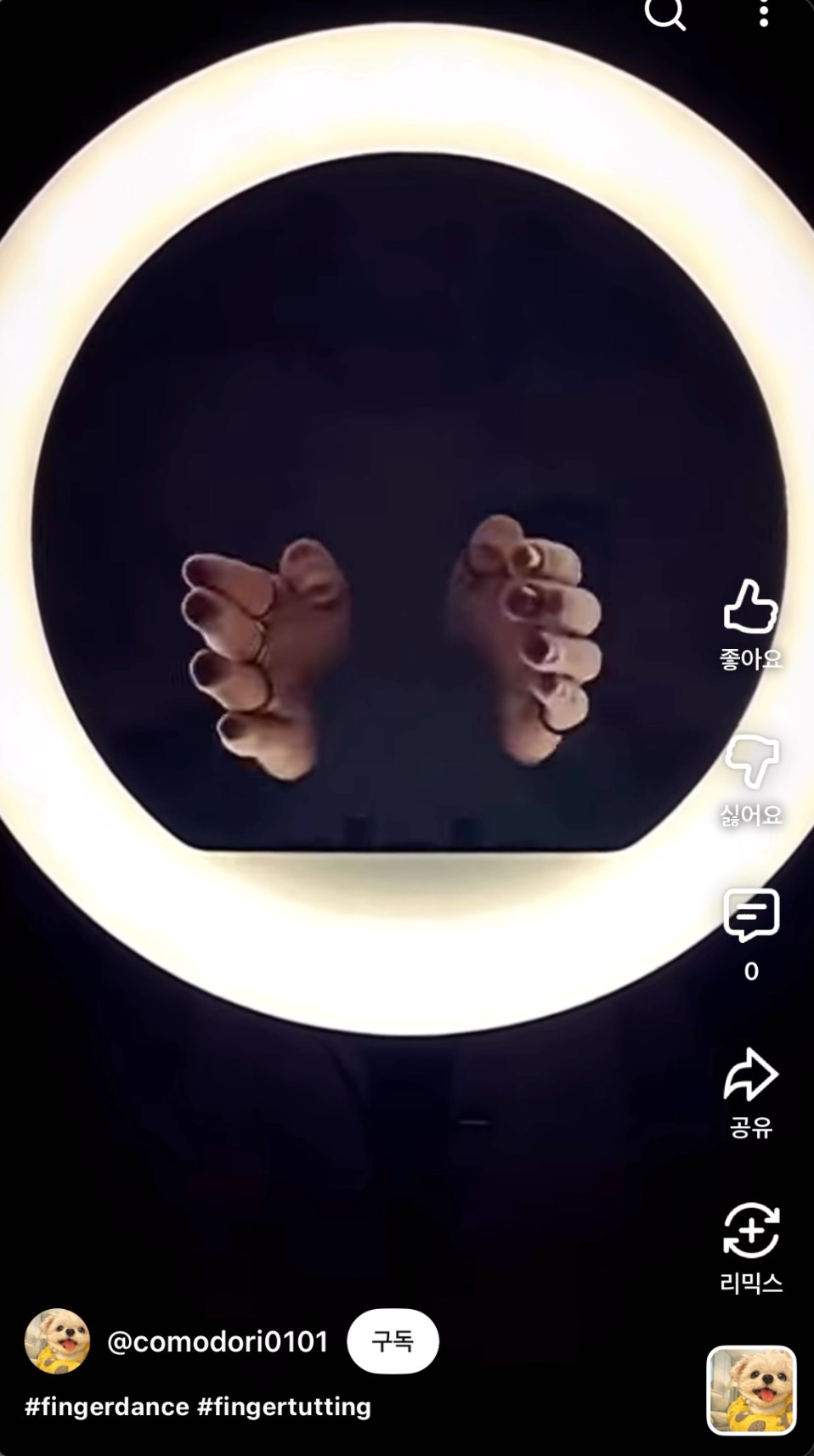


**Supplementary Figure 4.**
**No-label condition for the hedonic stimulus.** Screenshots from an Instagram Reel by @kamon__7, posted to Instagram on March 3, 2023, showing the stimulus presented without a provenance label. Adapted with permission and used in this article by permission of the copyright holder.


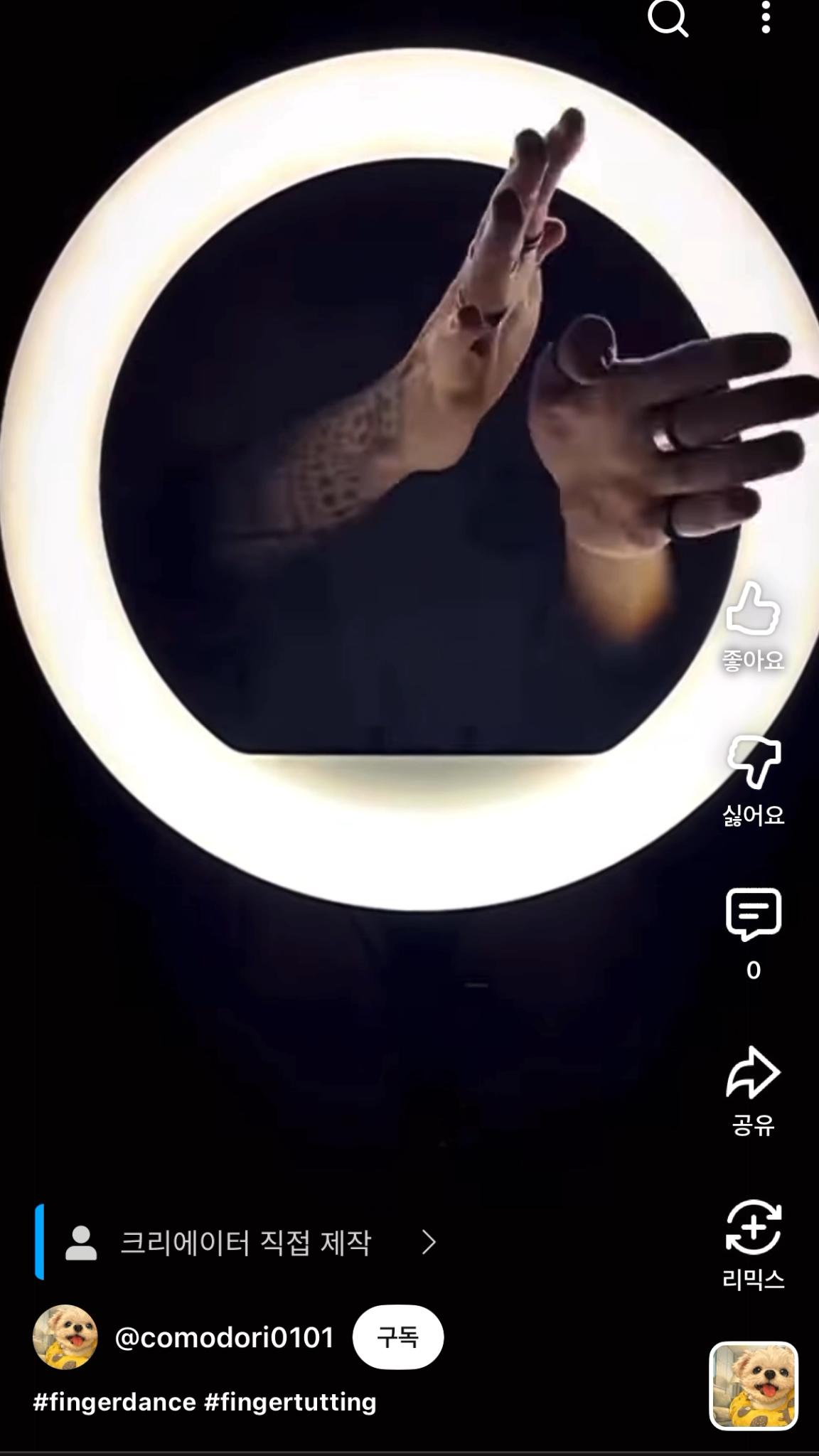

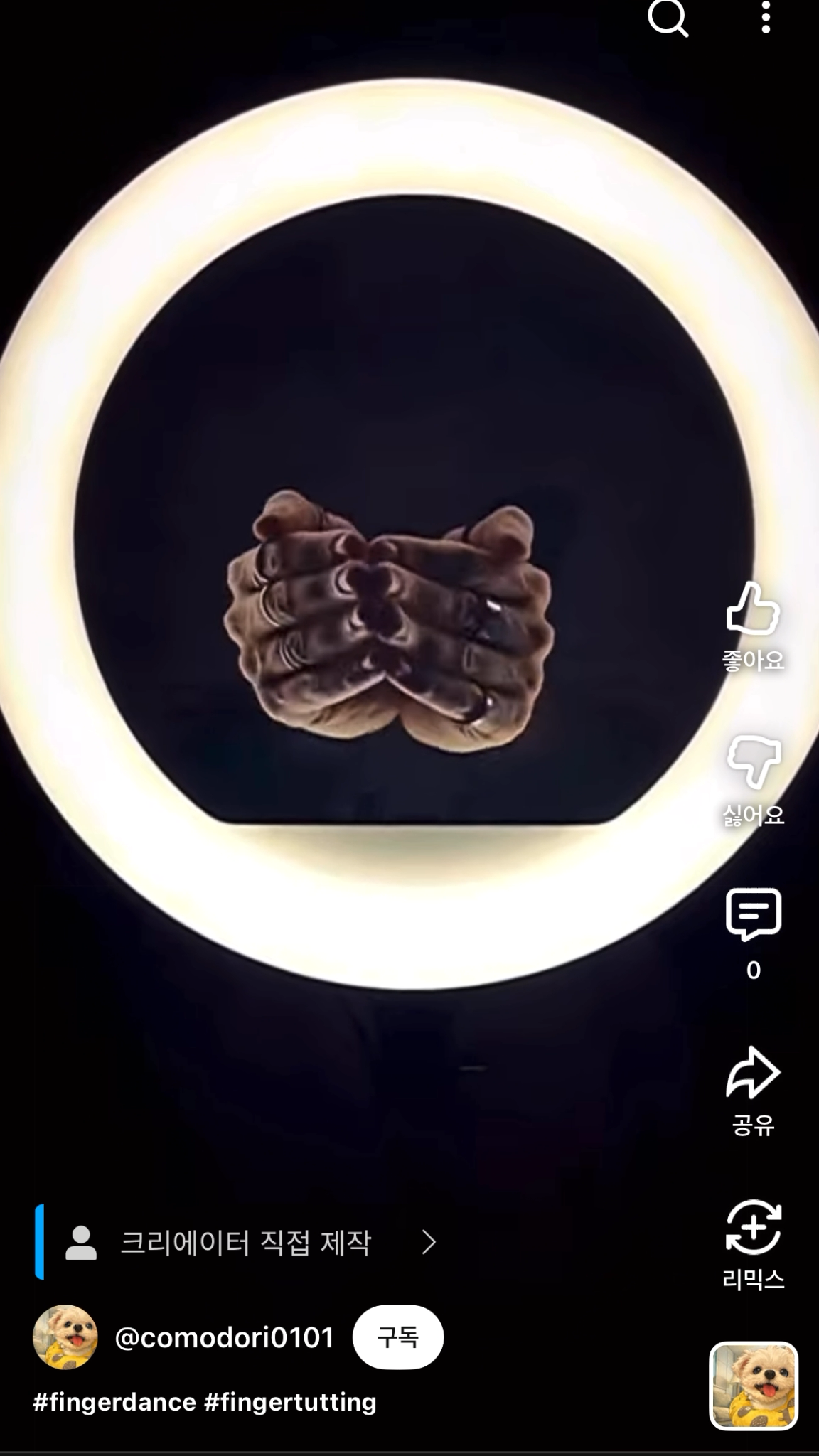

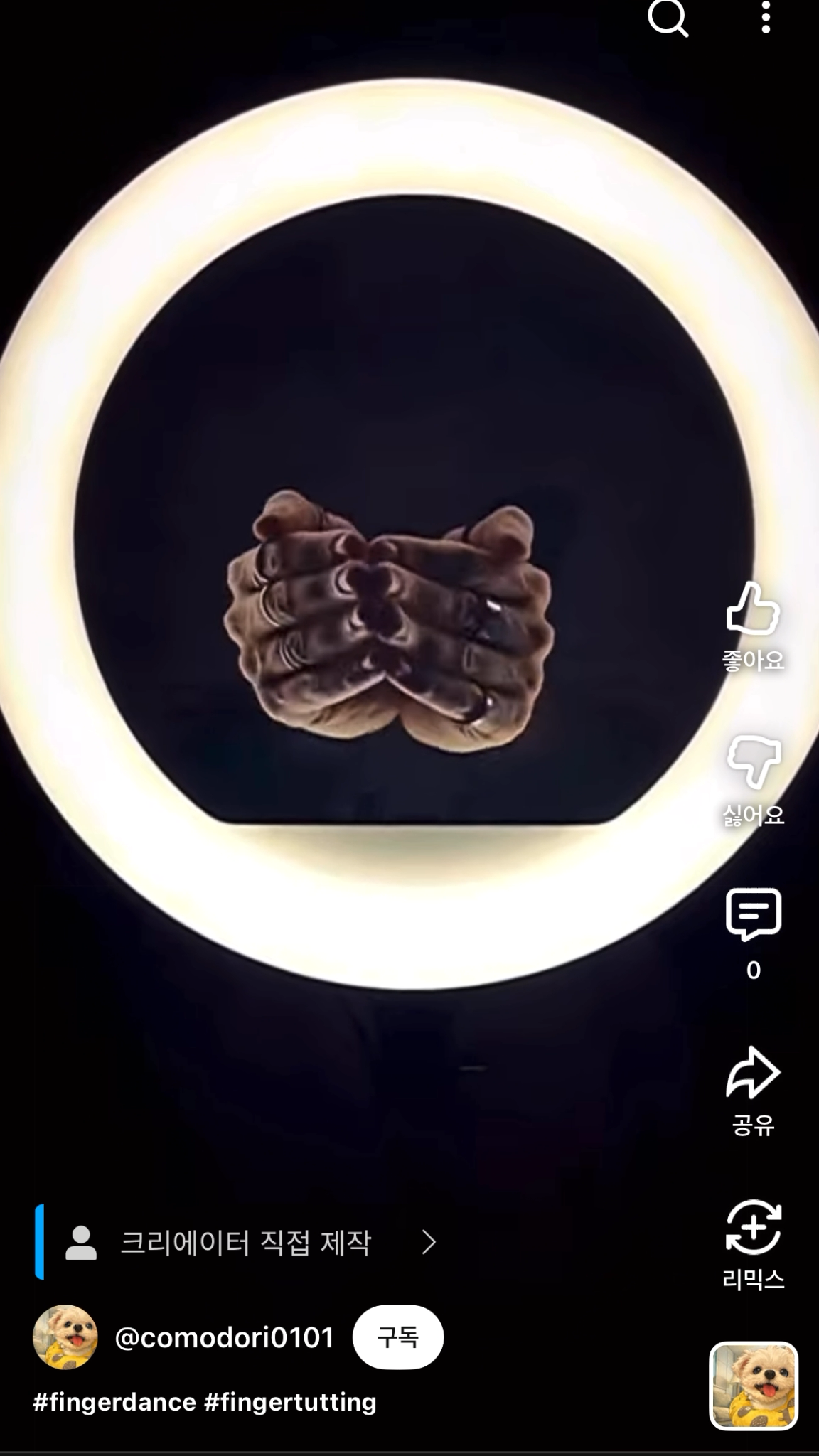


**Supplementary Figure 5.**
**Human-made label condition for the hedonic stimulus.** Screenshots from an Instagram Reel by @kamon__7, posted to Instagram on March 3, 2023, showing the stimulus presented with a Human-made provenance label. Adapted with permission and used in this article by permission of the copyright holder.


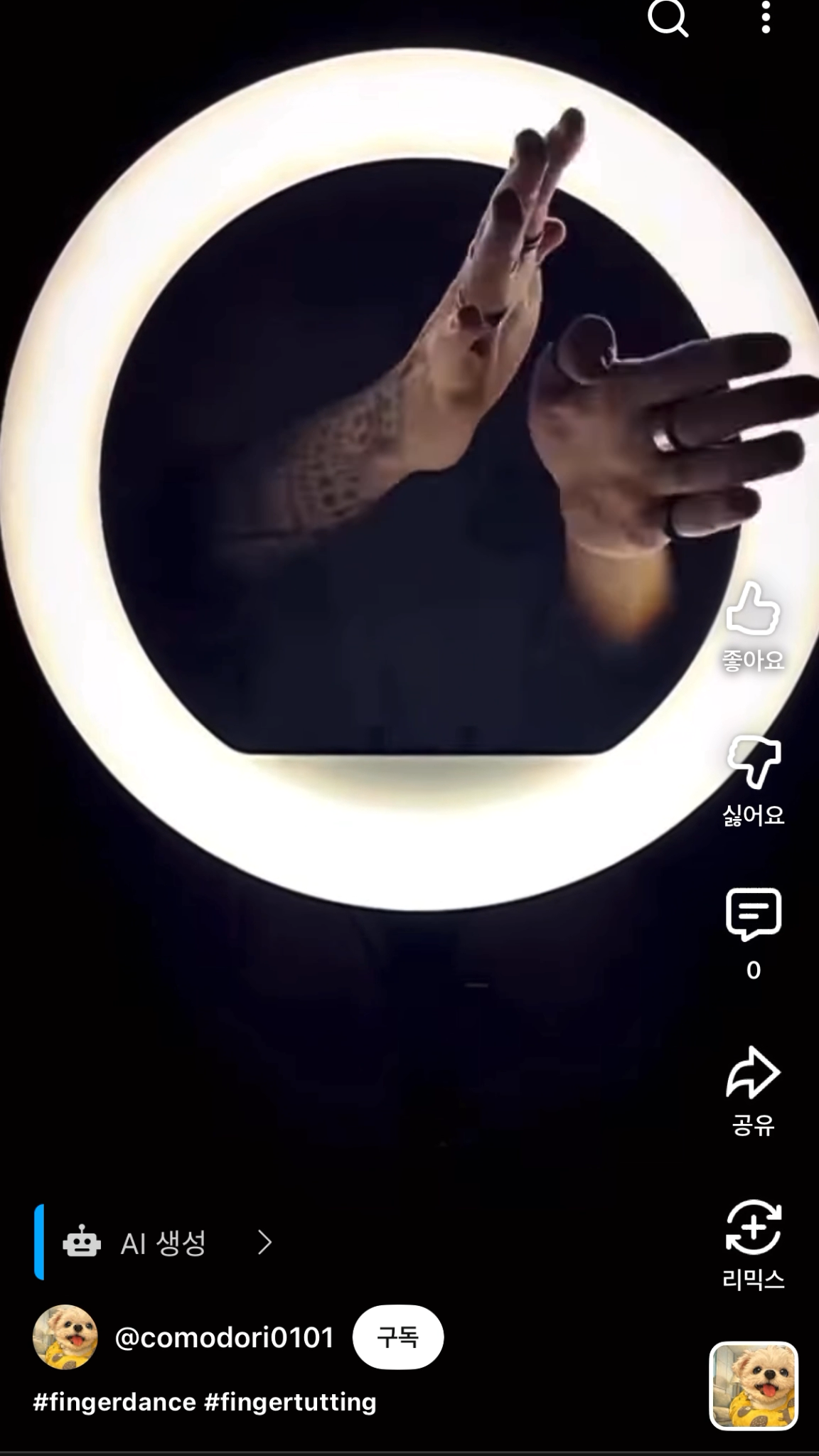

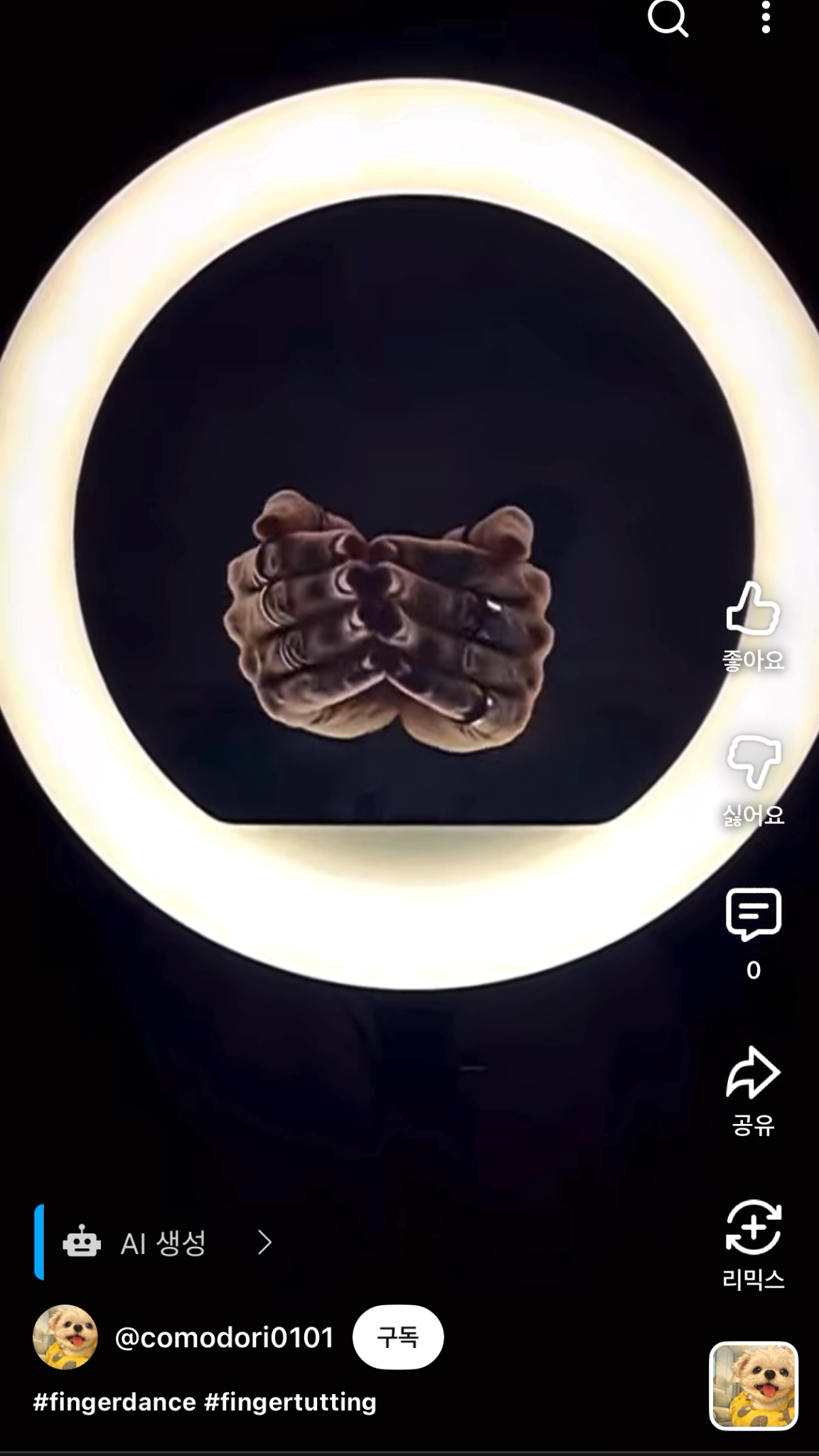

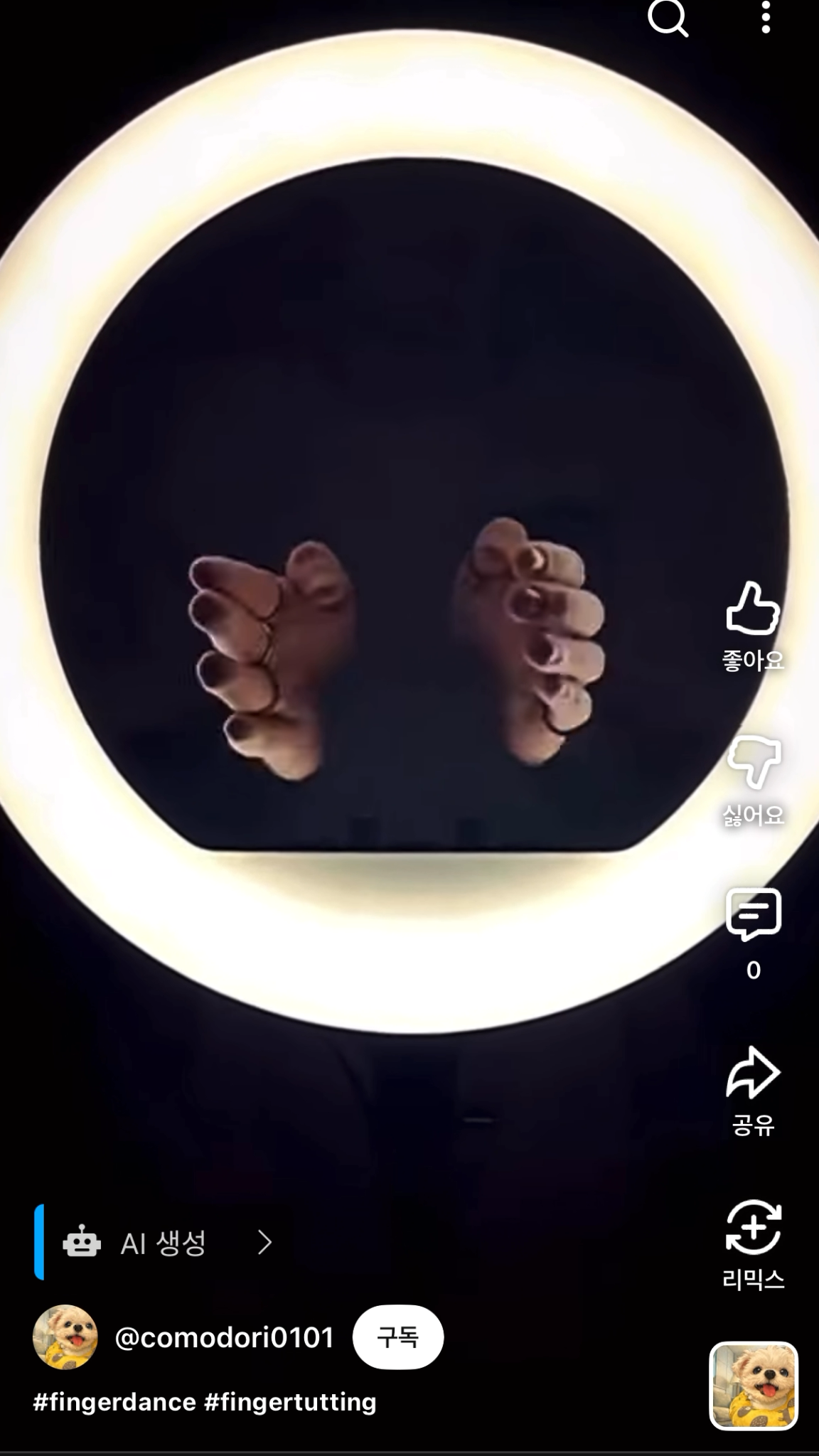


**Supplementary Figure 6.**
**AI-generated label condition for the hedonic stimulus.** Screenshots from an Instagram Reel by @kamon__7, posted to Instagram on March 3, 2023, showing the stimulus presented with an AI-generated provenance label. Adapted with permission and used in this article by permission of the copyright holder.
